# Host microbiome responses to the Snake Fungal Disease pathogen (*Ophidiomyces ophidiicola*) are driven by changes in microbial richness

**DOI:** 10.1038/s41598-022-07042-5

**Published:** 2022-02-23

**Authors:** Alexander S. Romer, Joshua B. Grinath, Kylie C. Moe, Donald M. Walker

**Affiliations:** 1 Department of Biology, Middle Tennessee State University, 1301 E Main St, Murfreesboro, TN 37132 USA; 2 Department of Biological Sciences, Idaho State University, 921 S. 8th Ave, Pocatello, ID 83209 USA

**Keywords:** Pathogens, Community ecology, Microbial ecology, Molecular ecology

## Abstract

Dermatophytic pathogens are a source of disturbance to the host microbiome, but the temporal progression of these disturbances is unclear. Here, we determined how Snake Fungal Disease, caused by *Ophidiomyces ophidiicola*, resulted in disturbance to the host microbiome. To assess disease effects on the microbiome, 22 Common Watersnakes (*Nerodia sipedon*) were collected and half were inoculated with *O. ophidiicola.* Epidermal swabs were collected weekly for use in microbiome and pathogen load characterization. For the inoculated treatment only, we found a significant effect of disease progression on microbial richness and Shannon diversity consistent with the intermediate disturbance hypothesis. When explicitly accounting for differences in assemblage richness, we found that β-diversity among snakes was significantly affected by the interaction of time and treatment group, with assemblages becoming more dissimilar across time in the inoculated, but not the control group. Also, differences between treatments in average microbiome composition became greater with time, but this interactive effect was not evident when accounting for assemblage richness. These results suggest that changes in composition of the host microbiome associated with disease largely occur due to changes in microbial richness related to disease progression.

## Introduction

Emerging infectious diseases (EIDs) have been identified by conservation biologists as a leading threat to global biodiversity in this century^1–3^. EIDs caused by fungal pathogens are of particular concern as they are more likely to result in the extinction/extirpation of their hosts when compared to diseases caused by other infectious agents^4^. Notable fungal diseases of wildlife include White-nose Syndrome of bats, Chytridiomycosis of amphibians, and Snake Fungal Disease^5–7^. Snake Fungal Disease (SFD) occurs in wild snake populations of many species, across a large geographic area in the United States, and has also been detected in numerous other countries^8–13^. Research has demonstrated that the fungus *Ophidiomyces ophidiicola* (previously *Ophidiomyces ophiodiicola* [Guarro, Deanna A. Sutton, Wickes and Rajeev] Sigler, Hambl. and Paré), is the causative agent of SFD^5,14^. Understanding host responses to EIDs, such as SFD, may largely depend on our knowledge of how pathogens interact with host microbiomes.

The tissues and organs of multicellular organisms provide dynamic habitat for the host microbiome^15,16^. The epidermis of vertebrates harbors a diverse assemblage of microbes whose composition can be altered by factors such as microtopography, host demographics, and environmental conditions^17^. Additionally, multicellular organisms possess complex relationships with their microbiome which can influence digestion, fitness, and pathogen susceptibility^18–20^. For example, Chytridiomycosis is known to alter the amphibian microbiome in wild populations and laboratory experiments^21^. Previous work has demonstrated that the epidermal microbiome of snakes sampled in the Eastern United States is distinct from environmental microbial assemblages^22^. Furthermore, host species, host habitat, and the presence of *O. ophidiicola* are predictive of the snake microbiome across spatial scales^22,23^. This suggests that the epidermal snake microbiome is not simply a product of random dispersal of microbes from the environment and is sensitive to host disease state. However, microbiome responses during disease progression are generally unclear. This study investigates the relationship between Snake Fungal Disease progression and disturbance to the skin microbiome within an ecological context.

Ecological disturbance can be defined as the alteration of an ecological system by a perturbing biotic or abiotic process^24^. Host microbial assemblages are subject to ecological disturbance including such processes as epidermal disease flares^25^. Consequently, it may be reasonable to conceptualize colonization of snake skin by a fungal pathogen, like *O. ophidiicola,* as a disturbance to the host microbiome. Disturbance is associated with increased variability in microbial assemblage composition and alterations to the relative importance of stochastic/deterministic assembly processes^26^. Additionally, increases in the magnitude or frequency of disturbance can result in changes to assemblage richness or other measures of diversity^27^. A widely accepted model relating disturbance and species richness is the intermediate disturbance hypothesis^28^. This hypothesis suggests that higher levels of disturbance will increase species richness, until a threshold value is reached, at which point additional levels of disturbance will decrease richness^28^. Given that the extent of clinical signs associated with SFD generally increases over time^14^, it is likely that the microbiome may be differentially affected at different stages of infection. However, it is unclear whether microbiome response to disease is consistent with the intermediate disturbance hypothesis or other patterns of change.

Many field studies examining SFD have used samples of snakes collected at a single time point^22,23,29,30^. However, field studies with repeat sampling report significant variability in clinical signs and fungal load within individuals^31–33^. Understanding the mechanisms that underlie disease state variability is an unresolved but central issue in wildlife disease. SFD has been correlated with negative impacts to snake overwintering and reproductive suppression^33–35^. However, changes in the host microbiome may, in part, explain variation in pathogen load and disease signs, as the microbiome serves as the first line of defense against pathogens^17^. Studies have shown that infection with *O. ophidiicola* alters the composition of the host microbiome^22,23^. Furthermore, some culturable skin microbes of snakes are known to have inhibitory effects against *O. ophidiicola*^36^. Thus, understanding the effects of fungi on the microbiome, at the skin interface, may inform our understanding of microbial response to a pathogen mediated environment.

The overall objective of this experiment was to determine the effects of *O. ophidiicola* on the host microbiome over temporal scales relevant to disease progression. We tested the following predictions: pathogen load will increase through time, prior to mortality, and infection will alter measures of alpha diversity (Operational Taxonomic Unit [OTU] richness, Shannon diversity, Shannon evenness), beta diversity (multivariate dispersion), and average community composition through time. To test these predictions, we inoculated snakes under controlled conditions and evaluated changes in microbial assemblages using metrics based on both the presence and abundance of OTUs to understand the community properties (i.e. richness, evenness) driving the observed patterns. This experiment provides a framework for interpreting the effects of wildlife pathogens on epidermal microbiomes over the course of disease progression. Additionally, our results elucidate bacterial-fungal interactions in a non-mammalian epidermal microbiome within the context of host disease and wildlife conservation.

## Results

We collected and analyzed 144 epidermal swabs via qPCR and high-throughput 16S rRNA amplicon sequencing. Eighteen snakes were swabbed on a weekly basis throughout the course of the experiment which lasted a total of 13 weeks. Eleven snakes were assigned to the inoculated treatment group and seven snakes were assigned to the sham treatment group. Over the course of the experiment, six inoculated snakes and four sham snakes died. Inoculated snakes had a mean value of 52% positive qPCR reactions when analyzed on a per-animal basis (i.e., not lumped before analysis). All snakes in the inoculated treatment group developed clinical signs of disease^37^.

### Pathogen load

**Figure 1:**
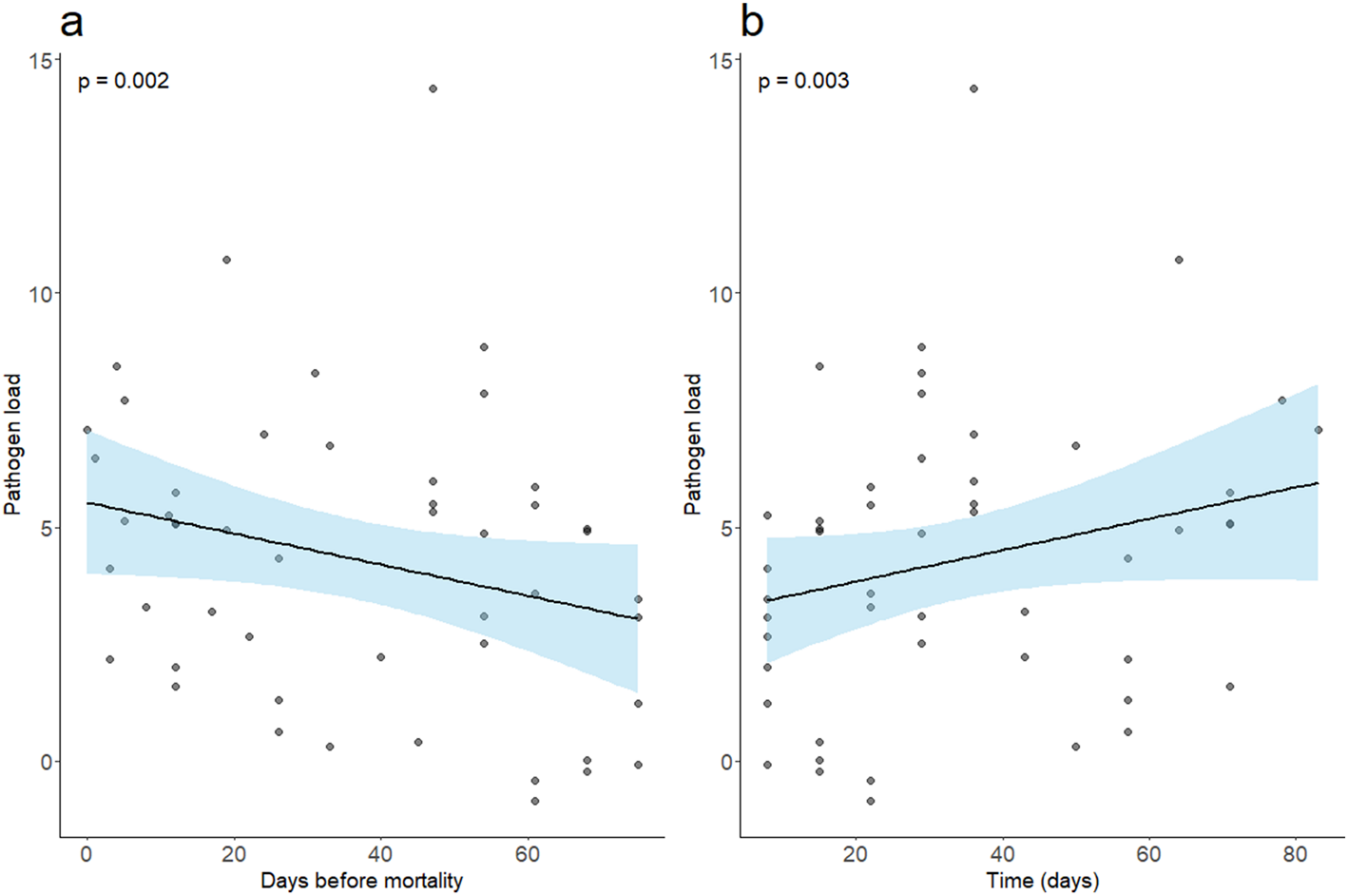
Days before mortality and time are predictive of pathogen load in inoculated snakes. (**a**) Pathogen load is represented as a function of time measured in units of days before mortality. Pathogen load values were derived from the natural log transformation of copy number values for *O. ophidiicola*. ﻿The negative trendline in this subplot indicates that as snakes were temporally closer to mortality, we observed higher pathogen loads. (**b**) The positive trendline in this subplot indicates that as the experiment progressed, we observed higher pathogen loads. LME model *p*-values for temporal effects are provided within each panel.

Days prior to mortality was found to be significantly predictive of copy number (LME, χ^2^ = 3.92, *p* = 0.002; Fig. 1a). Thus, as the days prior to an animal experiencing mortality decreased, copy number increased in qPCR positive swabs (slope = −0.04; SE = 0.02). Additionally, experimental time was found to be significantly predictive of copy number (LME, χ^2^ = 9.73, *p* = 0.003; Fig. 1b). Thus, as the experiment progressed, we detected higher pathogen load in our inoculated animals (slope = 0.07; SE = 0.03). These results indicate inoculation success and disease progression through time.

### Alpha diversity

**Figure 2:**
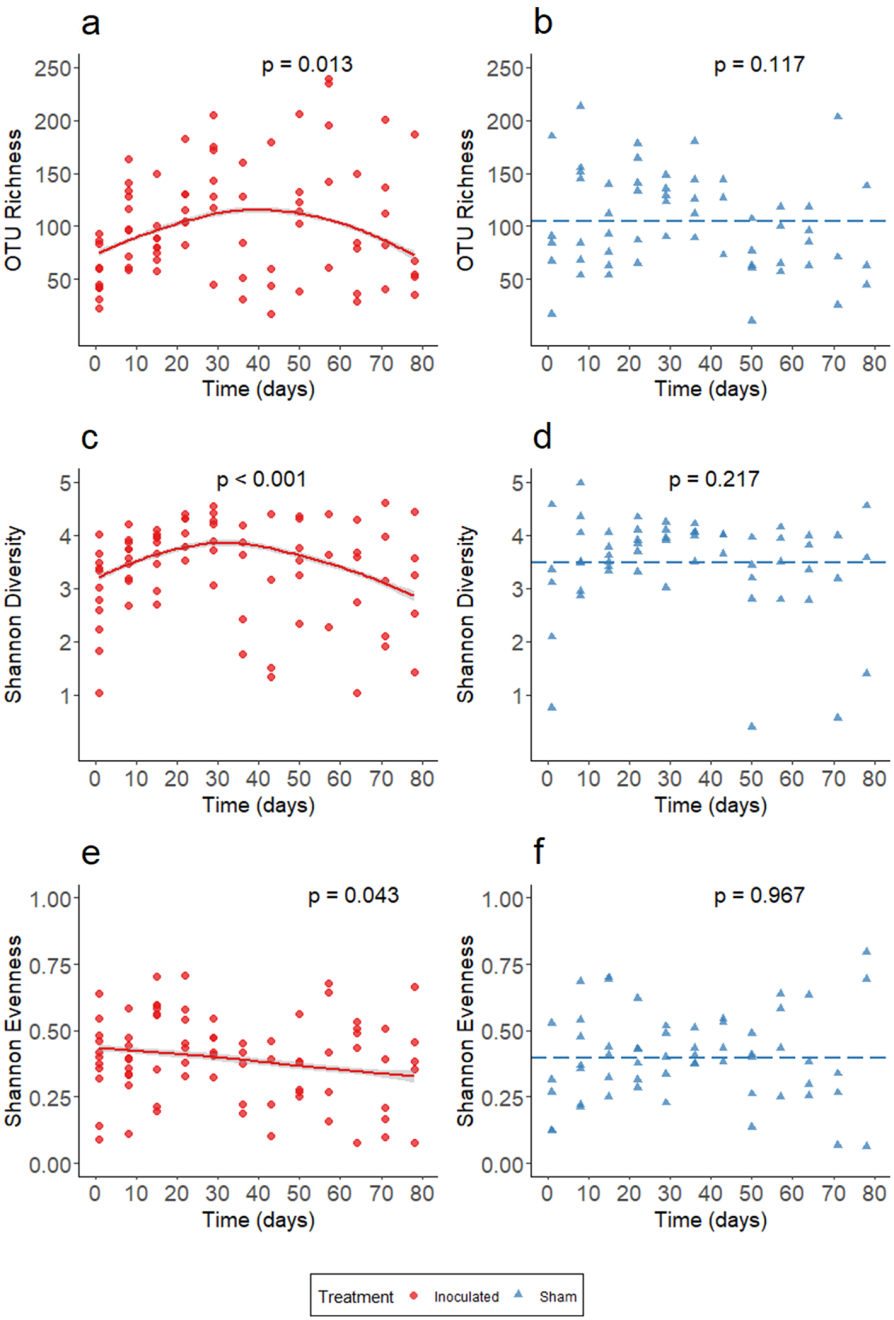
Time is predictive of OTU richness, Shannon diversity, and Shannon Evenness in inoculated but not sham snakes. (**a**,**b**) OTU richness of the host microbiome as a function of time. (**c**,**d**) Shannon diversity of the host microbiome as a function of time. (**e**,**f**) Shannon evenness of the host microbiome as a function of time. (**a**,**c**,**e**) Trendlines were generated using generalized additive mixed effects modeling. (**a**,**c**) Note that both OTU richness and Shannon diversity initially rose and then declined among the inoculated treatment group. (**e**) Among the inoculated treatment group, we observed a decrease in evenness through experimental time. (**b**,**d**,**f**) No significant effect of time was found for any measured component of alpha diversity among the sham control group. The dotted line in these subplots represents the mean value for OTU richness, Shannon diversity, or Shannon Evenness. *P*-values for temporal effects are provided within each panel.

A significant and non-linear trend for OTU richness was found for the inoculated treatment group through time (GAMM, edf = 2.53, F = 3.86, *p* = 0.0126; Fig. 2a) but not for the sham control group (GAMM, edf = 1.61, F = 1.83, *p* = 0.117; Fig. 2b). Inoculation produced a concave relationship where richness initially increased and then decreased through time. As with richness, a significant and non-linear trend for Shannon diversity (H) through time was observed for the inoculated treatment group (GAMM, edf = 2.559, χ^2^ = 20.75, *p* < 0.001; Fig. 2c) but not the sham control group (GAMM, edf = 2.18, χ^2^ = 2.82, *p* = 0.217; Fig. 2d). Thus, inoculation produced a non-linear trend on alpha diversity of the host microbiome, which was not observed in the sham control group. Furthermore, a significant effect of time on Shannon evenness (E) was observed for the inoculated treatment group (GAMM, edf = 1.00, F = 4.181, *p* = 0.043; Fig. 2e) but not the sham treatment group (GAMM, edf = 1.000, F = 0.002, *p* = 0.967; Fig. 2f). We observed a consistent decrease in evenness among inoculated snakes through experimental time. Additionally, as four of seven snakes in the sham control group died during the experiment, we assessed if mortality type (natural death or euthanasia) had a significant effect on alpha diversity among sham snakes. Time had a significant effect on OTU richness (GAMM, edf = 2.33, F = 4.058, *p* = 0.011) and Shannon diversity (GAMM, edf = 2.36, χ^2^ = 14.605, *p* = 0.001) among sham snakes that died naturally (Supplemental Material, Fig. S1). These relationships were concave in shape, but they did not drive patterns found when considering all sham snakes together in the analyses above. All results in this study were robust to the inclusion of mortality type as a covariate.

### β-diversity

**Figure 3:**
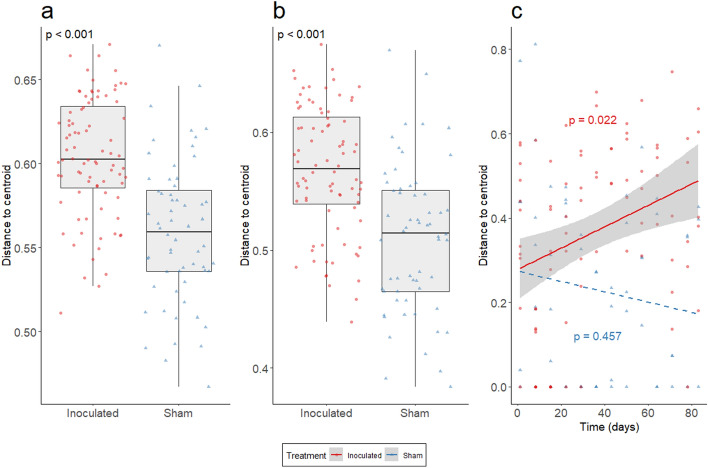
Inoculation results in higher β-diversity of the host microbiome. Multivariate dispersion of the host microbiome was approximated by generating distance-to-centroid values for the (**a**) Jaccard, (**b**) Bray–Curtis, and (**c**) Raup-Crick dissimilarity metrics. (**a**,**b**) There was a significant difference between treatment groups for both the Jaccard and Bray–Curtis indices (boxplots; LME *p*-values within each panel). Inoculated snakes were predicted to have higher distance-to-centroid values indicating greater dissimilarity or β-diversity throughout the experiment. (**c**) A positive relationship was observed between time and distance-to-centroid values for the Raup-Crick metric in the inoculated treatment group. Thus, the host microbiome of inoculated snakes tended to become more dissimilar over time. No relationship between time and distance-to-centroid values was found for the Raup-Crick metric for the sham control group. Consequently, a dotted line was used to represent the regression for this group (*p*-values are provided for post-hoc regressions).

Multivariate dispersion of the pathogen inoculated group was significantly higher than the sham control group for both the Jaccard index (LME, χ^2^ = 32.49, *p* < 0.001; Fig. 3a, Table 1) and Bray–Curtis index (LME, χ^2^ = 23.69, *p* < 0.001; Fig. 3b, Table 1). The interaction of time and treatment group had a significant effect on multivariate dispersion of the Raup-Crick metric (LME, χ^2^ = 3.90, *p* = 0.048; Fig. 3c, Table 1). A post hoc assessment found that time had no significant effect on multivariate dispersion of the Raup-Crick metric for the sham control group (GLS, T-value = −0.75, *p* = 0.457; Fig. 3c) but did have a significant effect on inoculated treatment group (GLS, T-value = 2.34, *p* = 0.022; Fig. 3c). We observed a positive relationship between time and distance-to-centroid values for the Raup-Crick metric in the inoculated treatment group (slope = 0.002, SE = 0.001). The Raup-Crick metric is a presence/absence community dissimilarity metric, which generates a null expectation for the number of shared species between communities by relating global site occupancy of taxa to local site occupancy probabilities, and then accounting for sampling bias likely to occur due to differences in richness between sites^38^. Thus, when we explicitly account for differences in richness, pathogen inoculated snake microbiomes became more dissimilar over the entire course of the experiment resulting in increased β-diversity among snakes.

**Table 1:** Summary of LME models of multivariate dispersion.

	Fixed effects	Denominator degrees of freedom	Fixed effects estimates	Standard error of estimates	χ^2^	*P*-values	R-squared
Jaccard	Treatment	15	0.0235	0.0066	32.493	< 0.001	0.360
	Time	124	−0.0003	0.0002	4.278	0.039	
	Treatment*time	15	< 0.0001	0.0001	< 0.001	0.999	
	Mortality type	124	−0.0082	0.0046	3.200	0.074	
Bray–Curtis	Treatment	15	0.0329	0.0105	23.694	< 0.001	0.279
	Time	124	−0.0001	0.0003	0.102	0.749	
	Treatment*time	15	−0.0001	0.0002	0.045	0.832	
	Mortality type	124	−0.0125	0.0071	3.066	0.080	
Raup-Crick	Treatment	15	0.0098	0.0388	8.698	0.003	0.231
	Time	124	0.0008	0.0009	1.539	0.215	
	Treatment*time	15	0.0017	0.0009	3.900	0.048	
	Mortality type	124	−0.0173	0.0265	0.423	0.515	

Each subsection represents a distinct community dissimilarity metric (Jaccard, Bray–Curtis, and Raup-Crick, respectively). Predictor variables included in the model are denoted in the Fixed effects column. All other columns detail model output including denominator degrees of freedoms, Wald chi-square values, and *p*-values for each fixed effect. Evaluation of the LMER models was conducted using type-II sum of squares.

### Assemblage composition

**Figure 4:**
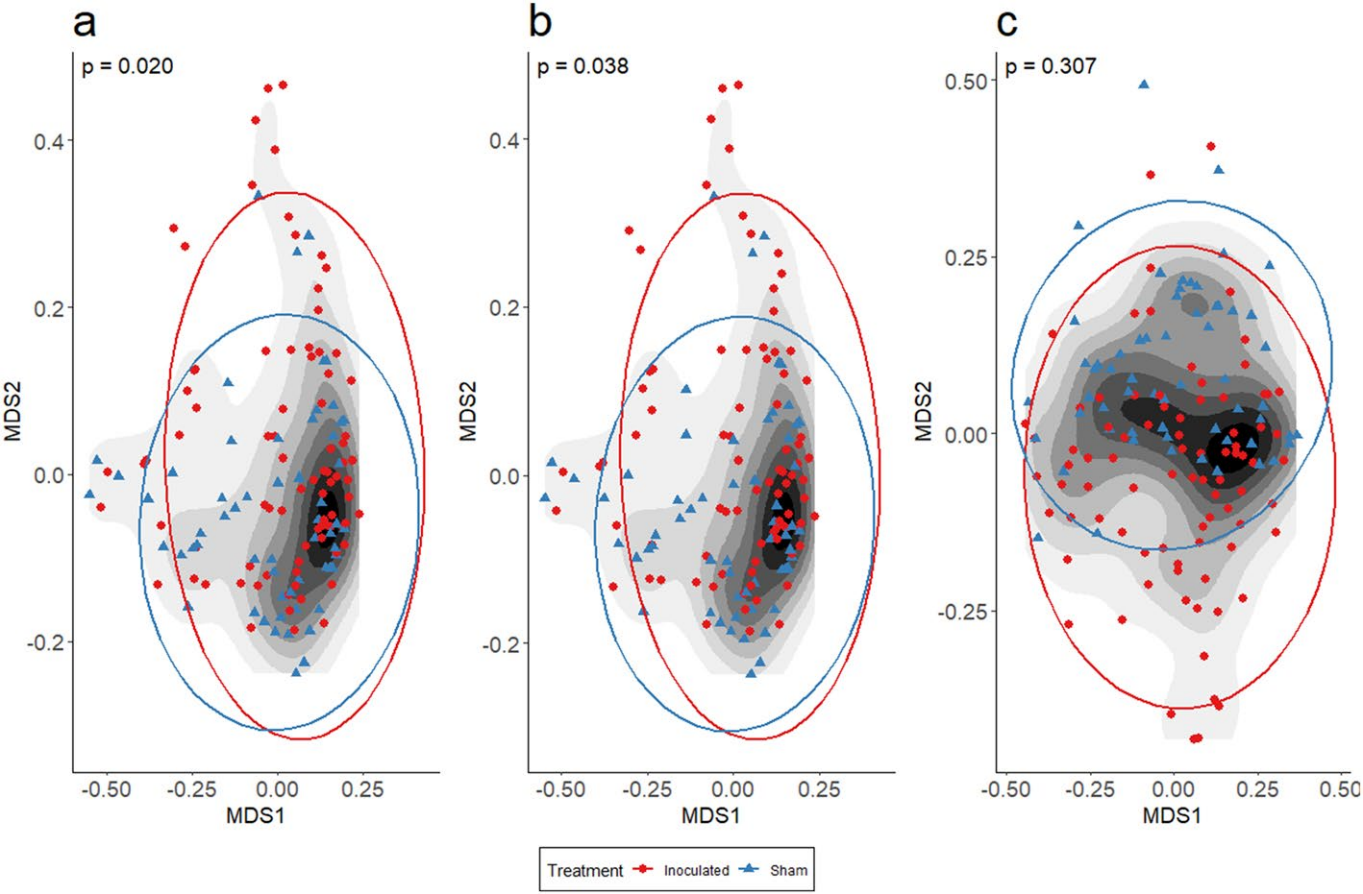
Inoculation results in changes to host microbiome composition which are partially explained by differences in richness. Nonmetric multidimensional scaling (NMDS) ordinations representing host microbiome composition of sham and inoculated snakes throughout the experiment measured using (**a**) Jaccard, (**b**) Bray–Curtis, and (**c**) Raup-crick dissimilarity. Multiple indices were used in order to discern patterns that were shared, or not, across indices. Differences in composition between sham and inoculated treatments are indicated by ellipses (95% SE); time is represented by contours with lighter colors corresponding to later sampling events (weeks). (**a**, **b**) The interaction of time and treatment group (PERMANOVA *p*-values within each panel) was significantly predictive of composition for both the Jaccard and Bray–Curtis index, suggesting that average composition of the host microbiome varied depending on treatment across time. (**c**) The interaction term was not significantly predictive of community composition for the Raup-Crick metric, which explicitly accounts for differences in composition that are likely to occur due to differences in richness alone. Changes in richness following infection with *O. ophidiicola* may be a key mechanism resulting in alterations to the host microbiome.

The interaction between experimental treatment and time was found to have a significant effect on the average microbial composition when measured using both the Jaccard index (PERMANOVA, F-stat = 1.34, *p* = 0.020; Fig. 4a, Table 2) and the Bray–Curtis index (PERMANOVA, F-stat = 1.44, *p* = 0.038; Fig. 4b, Table 2) but not the Raup-Crick metric (PERMANOVA, F-stat = 1.58, *p* = 0.307; Fig. 4c, Table 2). This suggests that differences in host microbiome composition between treatment groups are primarily explained by differences in OTU richness.

**Table 2:** Summary of PERMANOVA models of multivariate centroid position.

	Fixed effects	Degrees of freedom	Sum of squares	Partial R-squared	Mean squares	F-statistic	*P*-value
Jaccard	Treatment	1	0.91	0.014	0.905	2.13	0.001
	Time	1	1.34	0.021	0.13	3.15	0.001
	Treatment*time	1	0.57	0.009	0.569	1.34	0.020
	Mortality type	1	0.67	0.107	0.67	1.58	0.010
	Residuals	139	58.99	0.944	0.43		
	Total	143	62.46	1.000			
Bray–Curtis	Treatment	1	1.04	0.019	1.042	2.79	0.001
	Time	1	1.69	0.030	1.69	4.54	0.001
	Treatment*time	1	0.54	0.010	0.539	1.44	0.038
	Mortality type	1	0.70	0.013	0.70	1.88	0.008
	Residuals	139	51.83	0.929	0.38		
	Total	143	55.80	1.000			
Raup-Crick	Treatment	1	2.01	0.062	2.014	11.31	0.001
	Time	1	4.43	0.137	4.43	24.87	0.001
	Treatment*time	1	0.27	0.009	0.268	1.58	0.307
	Mortality type	1	0.90	0.028	0.90	5.08	0.008
	Residuals	139	24.76	0.764	0.18		
	Total	143	32.39	1.000			

Each subsection represents a distinct community dissimilarity metric (Jaccard, Bray–Curtis, and Raup-Crick respectively). Predictor variables included in the model are denoted in the Fixed effects column. All other columns detail model output, including F-statistics, partial R-squared, and *p*-values for each fixed effect.

## Discussion

Disturbance ecology allows investigators to predict and interpret the effects of perturbations, such as disease, on biotic assemblages^24^. In this investigation, *O. ophidiicola* was investigated as a potential ecological disturbance to the host microbiome. By inoculating snakes with *O. ophidiicola* in a controlled and pseudo-naturalistic setting, the effects of SFD could be measured through time for individual snakes. Thus, we were able to investigate temporal trends in host microbiome diversity and composition associated with infection. Our results suggest that SFD progression generates non-linear trends in host microbiome diversity. Furthermore, we found that analyses of null models of β-diversity (Raup-Crick dissimilarity) conflicted with traditional community dissimilarity metrics. This suggests that changes in assemblage richness are an important mechanism through which host microbiome composition is altered by *O. ophidiicola*. The patterns we observed within this system are consistent with conceptual models of disturbance ecology, in particular, the intermediate disturbance hypothesis.

Infection with *O. ophidiicola* can have detrimental consequences for the host such as increased basal metabolic rate and evaporative water loss^39^. Fungal pathogen load has been shown to increase at both the population and individual level over the course of an infectious disease outbreak^40,41^, which is consistent with our study results. We found that infection progressed in severity throughout the clinical trial for inoculated animals. While field studies do suggest chronic infection in free-roaming snakes^32–34^, other attempts to inoculate snakes with *O. ophidiicola* have reported a similar acute progression of disease as reported here^14,42^. Additionally, we found that higher pathogen load was associated with a shorter time until mortality. We were unable to determine that animals died from SFD via the methods used here as no necropsies were conducted, however, this does suggest that snakes with increasing pathogen loads are more likely to be in poor health. Pathogen load dynamics are predicted to be an important factor in the persistence or extirpation of amphibian populations affected by Chytridiomycosis^43^. Thus, further research into pathogen load dynamics is likely to be informative in understanding SFD outbreaks and for conservation management decisions. We acknowledge the elevated attrition rate (6/11 inoculated and 4/7 sham snakes died prior to the end of the experiment) of Watersnakes in captivity (similar to Neuman-Lee et al., 2014) and suggest a larger sample size during future studies^44^. Wild-caught snakes were brought into standardized conditions in captivity to explicitly test for microbiome differences due to disease while retaining biologically relevant microbial assemblages. This transition may have caused stress in study snakes. Indeed, non-diseased snakes that died naturally had temporal patterns of microbial richness and Shannon diversity similar to diseased snakes. However, our results are robust to the inclusion of mortality type in the analyses (Supplementary Material, Figure S1). In a similar fashion to pathogen load, we were able to test for temporal trends in the snake microbiome resulting from disease.

The microbiome is linked to host health, including processes of innate immunity^17^. Additionally, changes in alpha diversity of the microbiome are associated with factors such as obesity^45^, the environment^46^, and disease^47^. In this investigation, time was found to have a significant effect on OTU richness of pathogen inoculated, but not control snakes. Assemblage richness is sensitive to many factors including ecological disturbance^48^. Two factors suggest that ecological disturbance is a likely scenario to describe mechanistic interactions between skin bacteria and *O. ophidiicola*. First, a relationship was only observed between time and richness for the *O. ophidiicola* inoculated group. Previous work has shown that skin disease acts as a disturbance to the host microbiome resulting in changes to assemblage composition^25^. Second, the relationship between time (i.e., disease progression) and richness was concave for the *O. ophidiicola* inoculated group and absent for the sham control group. While the relationship between disturbance and richness varies depending on the ecological context, a widely accepted model relating these variables is the intermediate disturbance hypothesis. This hypothesis states that increasing levels of disturbance will increase species richness until a threshold value is reached, after which, species richness will decrease^28^. Our results are consistent with the intermediate disturbance hypothesis, as *O. ophidiicola* acts as a disturbance to the host microbiome, and disease progression leads to an increase in the magnitude of disturbance. It is likely this is a common process in host disturbed microbiomes, as pathogens are known to result in alternative stable states in frog skin microbiomes^49^ in both lab and field experiments^21^, and pathogens reduce the ability of the host or microbiome to regulate assemblage richness and/or composition^50^.

Previous observational studies of free-roaming snakes have demonstrated that the presence of *O. ophidiicola* correlates with alterations in the composition of the epidermal microbiome^22,23^. In this experiment, we implemented two traditional measures of community dissimilarity (Jaccard and Bray–Curtis) which are differentially sensitive to the effect of rare taxa. We found a significant interaction effect on average community composition, indicating that pathogen inoculation was predictive of host microbiome changes through time. We further investigated this pattern by applying the Raup-Crick index to our dataset and found no significant effect of the time × treatment interaction term on community composition. Together, these results suggest that differences in assemblage composition between pathogen inoculated and sham control snakes are described by differences in assemblage richness through time^51^.

To assess the relationship between disease progression and microbiome β-diversity we evaluated distance-to-centroid values representing the degree of heterogeneity in microbiome composition. The inoculated snake microbiome was found to have higher multivariate dispersion than sham snakes for both Jaccard and Bray–Curtis dissimilarity indices, indicating greater microbiome heterogeneity in the presence of a fungal pathogen. Additionally, multivariate dispersion of the Raup-Crick metric indicated that the sham snake microbiomes tended to be less variable, whereas, the pathogen inoculated microbiome increased in variability over time. This increase in dissimilarity occurred as microbial assemblage alpha diversity initially increased for diseased snakes, and then the assemblages continued to differentiate as taxa were lost, and alpha diversity (richness and Shannon diversity) decreased during advanced stages of disease progression. These changes were associated with decreased microbial assemblage evenness, indicating that some taxa became dominant, while others became rare through time. Our results indicate that disease progression generates increasing variability in host-microbiome composition even under conditions where the environmental microbial reservoir is similar. This suggests that fungal disease progression may be related to an increase in stochastic community assembly processes, such as drift, which can increase beta-diversity in the reptile microbiome^52^ as has been proposed in other animal microbiome systems^50^.

Wildlife diseases are an active area of research for understanding host-microbiome-pathogen interactions. This experiment has shown that the application of disturbance ecology can be useful in interpreting the effects of disease processes on the microbiome. We found that disturbance to the host microbiome occurred as a result of disease; causing alterations in alpha diversity. Our results show that *O. ophidiicola* alters the measured composition of the host microbiome throughout infection, but these changes are driven primarily by differences in richness, between healthy and diseased snakes. We found that SFD results in increased variability in the host microbiome with potential consequences for host health. We demonstrate that disruption of the host microbiome by a fungal pathogen is associated with host-health outcomes and changes to the host-associated epidermal microbiome. Given the growing threat of EIDs to wildlife in the twenty-first century, developing an increased understanding of the relationship between disease and microbiome ecology is crucial to inform effective conservation strategies.

## Methods

### Snake and soil collection

The Common Watersnake (*Nerodia sipedon*) is a nonvenomous semi-aquatic snake found throughout Eastern and Central North America^53^. We collected 22 N*. sipedon* in Tennessee during spring 2019. Any animal from the sham control group that had a positive qPCR reaction (Ct < 39) throughout the experiment (n = 4) was removed from all statistical analyses. Study snakes had a mean snout-vent length (SVL) of 28.4 cm and a mean mass of 11.4 g (Supplementary Material, Table S1). Study snakes were primarily neonates or juveniles with 15 of 18 animals having an SVL less than 25 cm (Supplementary Material, Table S1). We used nitrile gloves while handling snakes to prevent transmission of microbes between animals. We collected snakes free of clinical signs, to control for initial disease state, and further confirmed absence of *O. ophidiicola* using quantitative PCR (qPCR) of skin swabs^54^. We collected soil from snake capture locations to create a pseudo-naturalistic environmental reservoir of microbes. We collected a two-liter bag of soil three meters away from the closest riverbank of each snake capture location. As with snake samples, we confirmed the absence of *O. ophidiicola* in soil samples via qPCR. We stored soil samples at 4 °C in darkness in a bag that allowed for gas exchange but conserved moisture until mesocosm construction.

### Mesocosm design and animal care

Throughout the experiment, snakes were maintained individually in 66.24 L plastic storage totes (66 × 34 × 41 cm) with ventilation holes. We used a soil/aspen substrate mixture to provide an environmental reservoir of microbes. Specifically, equal parts, by weight, of each soil sample were mixed for 15 min until homogeneous. We combined this soil mixture with autoclaved aspen shavings in a 2:1 soil to aspen ratio by weight. We evenly layered this substrate into snake enclosures in a ~ 6 cm deep layer. When soiled, substrate was spot cleaned and replaced completely whenever snakes spilled large amounts of water. Snake enclosures had a hide box, autoclaved climbing branch, and water dish. Enclosures were located in a 23 °C room with a 12-h light/dark cycle. Depending on animal size, snakes were offered as many Guppies (*Poecilia reticulata*; small snakes; < 35 cm SVL) or Platies (*Xiphophorus maculatus*; large snakes; > 35 cm SVL) as they would consume weekly.

### Live animal trials

The experiment began on 31 May 2019 and concluded on 21 August 2019. Eleven snakes were randomly assigned to both the inoculation treatment and sham control groups. The snakes in the treatment group were inoculated with *O. ophidiicola* using the following procedure: A culture of *O. ophidiicola* was grown on Sabouraud dextrose agar (SDA) for 15 days and sectioned into 0.5 cm^2^ blocks then placed, mycelium side up, onto a waterproof bandage. Similar to Lorch et al. (2015), #150 sandpaper was used to abrade, via five strokes, the dorsal ventral and neck surface of the skin of each snake. Bandages with *O. ophidiicola* agar blocks were placed on each abrasion site to inoculate the skin for 72 h before being removed. Animals in the sham control group received the same treatment although sterile SDA blocks were applied to the bandages. Every seven days, samples were collected of the epidermal microbiome for all study snakes. Aseptic technique was used whenever work was conducted in and around the mesocosms to ensure that *O. ophidiicola* and other microbes were not transferred between enclosures. The swabbing protocol used to collect microbial samples involved wetting a rayon-tipped sterile applicator (Puritan 10808-146; VWR) with Millipore water that had been autoclaved for two hours. The applicator was then rolled using a stroking motion over a 15 cm portion of the snake’s midbody 15 times to standardize the sampled grain size^22,55^. All swab samples were stored at -20 °C until DNA extraction. This study was completed under IACUC MTSU-19-3012 approval and carried out according to relevant animal care and ARRIVE guidelines.

### Quantifying pathogen load

DNA was extracted from swab samples using the DNeasy PowerSoil kit (Qiagen) per the manufacturer’s protocol (*n* = 144 total samples). On each 96 well plate, a single DNA control blank was extracted to filter out contamination during qPCR and bioinformatic analyses. Pathogen load was measured using qPCR of the ITS gene marker of *O. ophidiicola*^54^. Quantitative PCR reactions and criteria for detection of positive samples followed the methods described in Walker et al.^22^. To determine pathogen load within each sample, a serial dilution of 1–1 × 10^10^ copies of a synthetic DNA fragment representing the qPCR target sequence (gBlock; Integrated DNA Technologies) was used to generate a standard curve.

### Amplicon sequencing and bioinformatics

To characterize microbial assemblages, a 250 bp region of the 16S rRNA marker was PCR amplified using primers 515F and 806R^56^. Amplicons were dual indexed following Fadrosh et al.^57^. Indexed amplicons were selected based on fragment size to remove adapter dimers using HighPrep magnetic beads (MagBio Genomics). The concentration of each library was quantified using a Quantus fluorometer (Promega), normalized, and pooled before sequencing on the Illumina MiSeq platform (2 × 250 bp paired end reads). Mothur v1.43.0 was used to conduct bioinformatic analyses according to the MiSeq SOP^58,59^ with several modifications. After forming contigs, *screen.seqs* was used to remove primers and barcodes. Sequences with a minimum of 248 bp and maximum length of 256 bp were then selected for downstream analysis. Sequences with ambiguous base calls and homopolymers greater than eight were removed from the data set. Remaining sequences were aligned to the SILVA v132 reference alignment^60,61^. The data were denoised using *pre.cluster* to merge sequences with two or fewer nucleotide differences. The *chimera.vsearch* command was used to remove chimeric sequences using the parameter ‘template = self’. Sequences identified as chloroplast, mitochondria, unknown, Archaea or Eukarya were removed from the dataset. Sequences were clustered into operational taxonomic units (OTUs) at 97% similarity using the *cluster.split* command. OTUs identified in negative control sequencing blanks (1602 total OTUs) were removed from the final data set. Rare OTUs (< 5) were removed using the *remove.rare* command and ‘bygroup = T’ option to remove any OTU that had fewer than the threshold sequences (< 5) on a per sample basis. Rarefaction of assemblages, based on total sequence reads, is typically conducted on a per sample basis in order to normalize ‘sampling effort’ across samples^62^. Therefore, we subsampled our data at 1102 sequence reads to generate a final rarefied abundance dataset that was imported into R v3.6.3 for statistical analysis^63^.

### Analysis of pathogen load

Analysis of qPCR data was restricted to positive samples from the inoculated treatment group. This allowed us to test hypotheses regarding changes in pathogen load. Specifically, we tested if time before an inoculated animal experienced mortality (natural or euthanasia) was predictive of *O. ophidiicola* copy number (fungal load). Modeling was accomplished using linear mixed effects models in *nlme*. We natural log transformed the copy number data to meet model assumptions of normality. We included days prior to mortality, mortality type (natural or euthanasia), and their interaction as fixed effects. Snake identity was included as a random effect and log copy number as the response variable. Death was defined as the date at which an individual experienced mortality over the clinical trial or was euthanized because the experiment concluded. Model fit was characterized using manual review of model residuals. Model selection was performed using Akaike Information Criterion (AIC) to determine if the inclusion of a random effect, temporal autocorrelation, and/or unequal variance term was appropriate^64^. A model was considered superior to another model iteration if the associated AIC value of that model was > 2 below the less complex model. In instances where there was not a difference of > 2, the simpler model was selected.

We also tested for the effect of experimental time on pathogen load. For this model, we included experimental time, mortality type, and their interaction as fixed effect terms, whereas log copy number was the response variable. Snake identity was included as a random effect. Model selection was performed as described above. Neither a temporal autocorrelation nor unequal variance term was indicated for inclusion in either model. Analysis of variance with type-II sum of squares (Wald χ^2^ statistics; *car* package) was used to determine the significance of the fixed effects while accounting for unequal sample sizes among snakes due to differential mortality^65^.

### Analysis of alpha diversity

We employed a generalized additive mixed effects model, via the function *gam* from the software package *mgcv*, to test if time had linear or non-linear effects on the richness of the host microbiome. Our model formula specified richness as the response variable. Time, treatment group, and mortality type (natural or euthanasia) were designated as fixed effects. The effect of time was modeled separately for each treatment group. Basis complexity (k) was limited to four to prevent model overfitting^66^. Random effects were specified using the ‘bs = re’ argument to the smoothing function. Akaike Information Criterion values were used to determine the most efficient random effects structure, distribution function, transformation link, and if the inclusion of a temporal autocorrelation term was appropriate^64^. Shannon-diversity (H) was calculated using the *diversity* function with the argument “index = shannon” in *vegan*. We then used a generalized additive mixed effects model in the same manner described above. Shannon-evenness index (E) was calculated by computing the exponential function (e^x^) of an assemblage’s Shannon-diversity and dividing that by the assemblage’s OTU richness^67^. In the same manner as the other diversity metrics, a generalized additive mixed effects model was used to make inferences regarding this metric. Since four of seven sham control snakes died during the experiment, we generated generalized additive mixed effects models in order to determine the effect of time on alpha diversity, according to mortality type (natural or euthanasia) within the sham treatment group. More specifically, we generated three models corresponding to each of the measures of alpha diversity examined in this study: OTU richness, Shannon diversity, and Shannon evenness. In each model, a measure of alpha diversity was specified as the response variable. Time and mortality type (natural or euthanasia) were designated as fixed effects. The effect of time was modeled as a smooth term separately for each level of mortality type. Basis complexity (k) was limited to four to prevent model overfitting. Random effects were specified using the ‘bs = re’ argument to the smoothing function. The function *gam.check* from the package *mgcv* was used to ensure that sufficient basis complexity was supplied to all fixed effects terms. Restricted maximum likelihood was used to estimate smoothing parameters by specifying “REML” to the *method* argument. Stepwise AIC selection, as described above, was used to determine inclusion of a random effect, alternative distribution, and/or temporal autocorrelation terms.

### Analysis of β-diversity

We applied the Jaccard, Bray–Curtis, and Raup-Crick dissimilarity indices metric to our dataset using the functions *vegdist* and *raupcrick* from the package *vegan*. The Jaccard index treats compositional data as presence/absence, and therefore, rare species have a greater effect on measured dissimilarity^51^. The Bray–Curtis index accounts for species abundance, and therefore, rare species have less of an effect on measured dissimilarity^51^. The Raup-Crick metric is a presence/absence community dissimilarity metric, which generates a null expectation for the number of shared species between communities by relating global site occupancy of taxa to local site occupancy probabilities, and then accounting for sampling bias likely to occur due to differences in richness between sites^38^. Using this metric, one is able to explicitly account for differences in richness on measured community dissimilarity^38^. The function *betadisper* in *vegan* was used to generate distance-to-centroid values for each of these community dissimilarity metrics. Communities were grouped by the interaction of experimental time and treatment group. Thus, a multivariate centroid against which distance-to-centroid values can be calculated was created for each treatment group separately at all time points. Distance-to-centroid values were then evaluated using linear mixed effects modeling with the function *lme* from the package *nlme*. Distance-to-centroid values for each dissimilarity metric were used as the response variable while the fixed effects structure of all three models consisted of treatment group, time, their interaction, and mortality type. Stepwise AIC selection, as described above, was used to determine if the inclusion of a random effect, temporal autocorrelation, and/or unequal variance term was appropriate. An analysis of variance with type-II sum of squares (Wald χ^2^ statistics; *car* package) was used to determine the significance of the fixed effects. A post-hoc assessment of any significant interaction terms was performed using the *gls* function from *nlme*. For post-hoc assessments, each treatment group was modeled independently to determine the relationship between multivariate dispersion and time on a per treatment basis.

### Analysis of assemblage composition

The function *adonis* in *vegan* was used to conduct Permutational Multivariate Analysis of Variance on distance matrices generated via the dissimilarity metrics above. The specified fixed effect terms included treatment group, time, their interaction, and mortality type. A statistically significant interaction term would indicate average microbiome composition of each treatment group changed in a disparate fashion through time. For all *adonis* models, animal ID was specified as a grouping variable (using “strata = ”) to constrain permutations and account for repeated measures (see Supplementary Material, Methods Section for additional detail on all methods).

## Supplementary Information

Supplementary Information 1.

Supplementary Information 2.

Supplementary Information 3.

Supplementary Information 4.

## Data Availability

The R code and data frame to reproduce the above analysis are included within the Supplemental Material. Raw data are available through the corresponding author upon request.
