# Supplementary material for: Host microbiome responses to the Snake Fungal Disease pathogen (Ophidiomyces ophidiicola) are driven by changes in microbial richness
Source: Sci Rep. 2022 Feb 23;12:3078. doi: 10.1038/s41598-022-07042-5 (PMC8866498; doi:10.1038/s41598-022-07042-5)
Supplement: Supplementary file 2 — Supplementary Information 2. [file 41598_2022_7042_MOESM2_ESM.docx]

| Animal ID | Capture Location | Capture date | Treatment Group | Initial SVL (cm) | Initial Weight (g) | Clinical Survival | Mortality date |
| --- | --- | --- | --- | --- | --- | --- | --- |
| e1 | Cheatham | 4/21/2019 | Inoculated | 15.8 | 9.38 | No | 6/3/2019 |
| e2 | Cheatham | 4/28/2019 | Inoculated | 17.2 | 5.99 | No | 6/10/2019 |
| e3 | Cheatham | 5/1/2019 | Inoculated | 19.1 | 8.96 | No | 7/29/2019 |
| e4 | Putnam | 5/7/2019 | Inoculated | 19.2 | 9.60 | No | 6/19/2019 |
| e5 | Putnam | 5/16/2019 | Inoculated | 19.7 | 3.88 | No | 6/29/2019 |
| e6 | Putnam | 5/19/2019 | Inoculated | 18.5 | 4.06 | Yes | 8/21/2019 |
| e7 | Putnam | 5/19/2019 | Inoculated | 21.4 | 5.98 | Yes | 8/21/2019 |
| e8 | Putnam | 5/22/2019 | Inoculated | 18.2 | 3.70 | Yes | 8/21/2019 |
| e9 | Rutherford | 5/22/2019 | Inoculated | 35.5 | 27.33 | Yes | 8/21/2019 |
| e10 | Rutherford | 5/24/2019 | Inoculated | 21.5 | 5.41 | Yes | 8/21/2019 |
| e11 | Rutherford | 5/24/2019 | Inoculated | 44.6 | 44.63 | No | 6/18/2019 |
| c1 | Cheatham | 4/21/2019 | sham | 18.1 | 3.43 | No | 6/12/2019 |
| c2 | Cheatham | 5/1/2019 | sham | 17.8 | 3.25 | No | 6/24/2019 |
| c5 | Putnam | 5/16/2019 | sham | 34.5 | 16.50 | No | 8/14/2019 |
| c7 | Putnam | 5/19/2019 | sham | 18.3 | 7.33 | Yes | 8/21/2019 |
| c9 | Rutherford | 5/24/2019 | sham | 23.5 | 13.68 | No | 7/20/2019 |
| c10 | Rutherford | 5/24/2019 | sham | 23.6 | 14.91 | Yes | 8/21/2019 |
| c11 | Rutherford | 5/24/2019 | sham | 23.2 | 18.00 | Yes | 8/21/2019 |

**Supplementary Table S1. Summary of animals used in this experiment.** Table includes initial morphometrics, capture location, and mortality date. All animals were collected between the dates of 4/21/2019 and 5/24/2019.


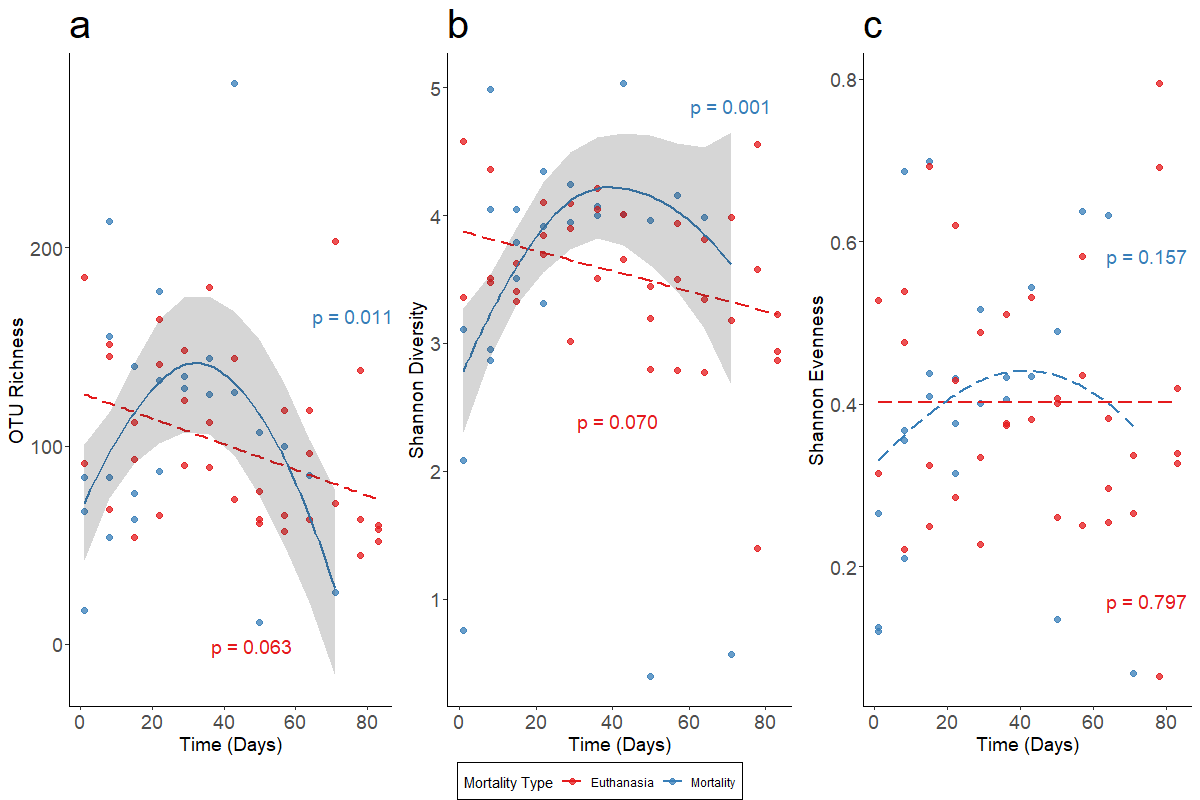

**Supplementary Figure S1. Effect of mortality type on alpha diversity in sham treatment.** (a) OTU richness of the host microbiome as a function of time. (b) Shannon diversity as a function of time (c) Shannon evenness as a function of time. (a,b,c) Trendlines were generated through the use of generalized additive mixed effect modeling. Time had a significant effect on OTU richness and Shannon diversity among sham snakes which experienced natural mortality. No significant effect of time was found for richness or diversity among sham snakes which survived until the end of the experiment. We observed no significant effect of time on Shannon evenness, regardless of mortality type, for sham snakes.

**Supplemental Methods**

***Snake & Soil Collection***

The Common Watersnake (*Nerodia sipedon*) is a nonvenomous semi-aquatic snake found throughout Eastern and Central North America^1^. We collected 22 *N. sipedon* in Tennessee during spring 2019 with TWRA approval (Scientific Collection Permit #1907). Study snakes had a mean snout-vent length (SVL) of 28.4 cm and a mean mass of 11.4 g (Supplementary Material, Table S1). Study snakes were primarily neonates or juveniles with 15 of 18 animals possessing an SVL less than 25 cm (Supplementary Material, Table S1). We used nitrile gloves while handling snakes to prevent transmission of microbes between animals. We collected snakes free of clinical signs, to control for initial disease state, and further confirmed absence of *O. ophidiicola* using quantitative PCR (qPCR) of skin swabs^2^. Collected snakes free of clinical signs were held for a maximum of 24 hours to allow for quantitative PCR (qPCR) detection of *O. ophidiicola*. Snakes’ positive for *O. ophidiicola* via qPCR were returned to their capture locations. We collected soil from snake capture locations to create a pseudo-naturalistic environmental reservoir of microbes. We collected a two-liter bag of soil three meters away from the closest riverbank of each snake capture location. As with snake samples, we confirmed the absence of *O. ophidiicola* in soil samples via qPCR. We stored soil samples at 4°C in darkness in a bag that allowed for gas exchange but conserved moisture until mesocosm construction.

***Mesocosm Design & Animal Care***

Throughout the course of this experiment, snakes were maintained individually in 66.24 L plastic storage totes (66 × 34 × 41 cm) with ventilation holes. We used a soil/aspen substrate mixture to provide an environmental reservoir of microbe. Specifically, equal parts, by weight, of each soil sample were mixed for 15 minutes until homogeneous. We then combined this soil mixture with autoclaved aspen snake bedding in a 2:1 soil to aspen ratio by weight. We evenly layered this substrate into snake enclosures in a ~6cm deep layer. When soiled, the substrate was spot cleaned and replaced completely whenever snakes spilled large amounts of water. Snake enclosures had a hide box, autoclaved climbing branch, and water dish. Enclosures were located in a 23°C room with a 12-hour light/dark cycle. Depending on animal size, snakes were offered as many Guppies (*Poecilia reticulata*; small snakes; < 35cm SVL) or Platies (*Xiphophorus maculatus*; large snakes; > 35cm SVL) as they would consume weekly.

***Live Animal Trials***

The experiment began on 31 May 2019 and concluded on 21 August 2019. Eleven snakes were randomly assigned to both the inoculation treatment and sham control groups. The snakes in the treatment group were inoculated with *O. ophidiicola* using the following procedure: A culture of *O. ophidiicola* was grown on Sabouraud dextrose agar (SDA) for 15 days and sectioned into 0.5 cm^2^ blocks then placed, mycelium side up, onto a waterproof bandage. Similar to Lorch et al. (2015), #150 sandpaper was used to abrade, via five strokes, the dorsal ventral and neck surface of the skin of each snake ^3^. Bandages with *O. ophidiicola* agar blocks were placed on each abrasion site to inoculate the skin for 72 hours before being removed. Animals in the sham control group received the same treatment although sterile SDA blocks were applied to the bandages. Every seven days, samples were collected of the epidermal microbiome for all 22 snakes. Aseptic technique was used whenever work was conducted in and around the mesocosms to ensure that *O. ophidiicola* and other microbes were not transferred between enclosures. The swabbing protocol used to collect microbial samples involved wetting a rayon-tipped sterile applicator (Puritan 10808-146; VWR; Radnor, PA, USA) with Millipore water (MilliporeSigma; Burlington, MA, USA) that had been autoclaved for two hours. The applicator was then rolled using a stroking motion over a 15 cm portion of the snake’s midbody 15 times to standardize the sampled grain size^4,5^. All swab samples were stored at -20°C until DNA extraction. Each snake was observed on a daily basis to perform general health checks. Euthanasia was accomplished at the end of the clinical trial by injection with sodium pentobarbital. This study was completed under IACUC MTSU-19-3012 approval and carried out according to relevant animal care and ARRIVE guidelines.

***Quantifying Pathogen Load***

DNA was extracted from swab samples using the DNeasy PowerSoil kit (Qiagen; Hilden, Germany) per the manufacturer’s protocol (n = 144 total samples). On each 96 well plate, a single DNA control blank was extracted to filter out contamination during qPCR and bioinformatic analyses. Pathogen load was measured using qPCR of the ITS gene marker of *O. ophidiicola* ^6^. Quantitative PCR reactions and criteria for detection of positive samples followed the methods described in Walker et al.^4^. Any animal from the sham control that had a positive qPCR reaction (C_t_ < 39) throughout the experiment (n = 4) was removed from all statistical analyses. To determine pathogen load within each sample, a serial dilution of 1 – 1 × 10^10^ copies of a synthetic DNA fragment representing the qPCR target sequence (gBlock; Integrated DNA Technologies; Coralville, IA, USA) was used to generate a standard curve. The log copy number of *O. ophidiicola* was calculated using the formula y = -0.2893x + 10.783, which was generated from the slope of standard curve C_t_ values. The value ‘x’ was the average Ct for each unknown sample run in triplicate.

***Amplicon Sequencing & Bioinformatics***

To characterize microbial assemblages, a 250 bp region of the 16S rRNA marker was PCR amplified using primers 515F and 806R^7^. Amplicons were dual indexed following Fadrosh et al.^8^. Indexed amplicons were selected based on fragment size to remove adapter dimers using HighPrep magnetic beads (MagBio Genomics; Gaithersburg, MD, USA). The concentration of each library was quantified using a Quantus fluorometer (Promega; Madison, WI, USA), normalized, and pooled before sequencing on the Illumina (San Diego, CA, USA) MiSeq platform (2 × 250 bp paired end reads). Mothur v1.43.0 was used to conduct bioinformatic analyses according to the MiSeq SOP^9,10^ with several modifications. After forming contigs, *screen.seqs* was used to remove primers and barcodes. Sequences with a minimum of 248 bp and maximum length of 256 bp were then selected for downstream analysis. Sequences with ambiguous base calls and homopolymers greater than eight were removed from the data set. Remaining sequences were aligned to the SILVA v132 reference alignment^11,12^. The data were denoised using *pre.cluster* to merge sequences with two or fewer nucleotide differences. The *chimera.vsearch* command was used to remove chimeric sequences using the parameter ‘template=self’. Sequences identified as chloroplast, mitochondria, unknown, Archaea or Eukarya were removed from the dataset. Sequences were clustered into operational taxonomic units (OTUs) at 97% similarity using the *cluster.split* command. OTUs identified in negative control sequencing blanks (1,602 total OTUs) were removed from the final data set. Rare OTUs (< 5) were removed using the *remove.rare* command and ‘bygroup=T’ option to remove any OTU that had fewer than the threshold sequences (< 5) on a per sample basis. Rarefaction of assemblages, based on total sequence reads, is typically conducted on a per sample basis in order to normalize ‘sampling effort’ across samples^13^. This procedure is necessary to ensure the accuracy of both univariate and multivariate descriptors of an assemblage^13–15^. Therefore, we subsampled our data at 1,102 sequence reads to generate a final rarefied abundance dataset that was imported into R v3.6.3 for statistical analysis^16^.

***Analysis of* *Pathogen Load***

Analysis of qPCR data was restricted to positive samples from the inoculated treatment group. This allowed us to test hypotheses regarding changes in pathogen load. Specifically, we tested if time before an inoculated animal experienced mortality (natural or euthanasia) was predictive of *O. ophidiicola* copy number (fungal load). Modeling was accomplished using a linear mixed-effects model in *nlme*^17^. We natural log transformed the copy number data to meet model assumptions of normality. We included days prior to mortality, mortality type, and the interaction between these variables as fixed effects. Snake identity was included as a random effect and log copy number was our response variable. Death was defined as the date at which an individual experienced mortality over the clinical trial or was euthanized because the experiment concluded. Model fit was characterized using manual review of model residuals. Model selection was performed using Akaike Information Criterion (AIC) values to determine if the inclusion of a temporal autocorrelation or unequal variance term was appropriate^18^. A model was considered superior to another model iteration if the associated AIC value of that model was > 2 below the less complex model. In instances where there was not a difference of >2, the simpler model was selected. Neither a temporal autocorrelation nor unequal variance term was indicated for inclusion in the model.

We also tested for the effect of experimental time on pathogen load. For this model, we included experimental time, mortality type, and the interaction between these variables as fixed effect terms, whereas log copy number was our response variable. Snake identity was included as a random effect. Experimental time was defined in units of days post-inoculation. Model selection was performed as described above. Neither a temporal autocorrelation nor unequal variance term was indicated for inclusion in the model. Analysis of variance with type-II sum of squares (Wald χ² statistics; *car* package) was used to determine the significance of the fixed effects while accounting for unequal sample sizes among snakes due to differential mortality^19^.

***Analysis of* *Alpha Diversity***

Microbiome data is multidimensional, thereby, necessitating the use of both univariate and multivariate measures of assemblage composition^13^. Several univariate measures including OTU richness and the myriad of alpha-diversity metrics can be used to fully describe observed variation^20^. Microbial community data were imported into R in the form of a sparse rarefied abundance table where individual OTUs were assigned to columns and an individual row was assigned to each sample. OTU abundance data was converted to presence/absence using the function *decostand* and the argument ‘method=pa’ in *vegan*^21^. Row sums were then calculated using the function *rowSums* to represent OTU richness in a given sample.

We employed a generalized additive mixed effect model, via the function *gam* from the software package *mgcv*, to test if time had linear or non-linear effects on the richness of the host microbiome^22^. Our model formula specified richness as the response variable. Time, treatment group, and mortality type (natural or euthanasia) were designated as fixed effects. Time was wrapped in the smoothing function (*s*) from *mgcv* which used the ‘by =’ argument to determine the effect of time separately for each treatment group. Basis complexity (k) of this fixed effect term was limited to four to prevent model overfitting^23^. Random effects were specified using the ‘bs = re’ argument to the smoothing function. Akaike Information Criterion values were used to determine the most efficient random effects structure, distribution function, transformation link, and if the inclusion of a temporal autocorrelation term was appropriate^18^. The most efficient model included measurement week as a random intercept. In a separate random effect term, animal ID was included as a random intercept and experimental time was included as a random slope. The model featured the gamma distribution family and included a log transformation link. A temporal autocorrelation term was not indicated for inclusion in the model.

Shannon-diversity (H) was calculated using the *diversity* function with the argument “index = shannon” in *vegan*^21^. We then used a generalized additive mixed effect model with the function *gam* from the software package *mgcv* in the same manner described above^22^. The most efficient model included animal ID as a random intercept, experimental time as a random slope, and featured the ‘scat’ distribution family from the package *mgcv*. Neither a temporal autocorrelation term nor a transformation link was indicated for inclusion in the model.

Shannon-evenness index (E) was calculated by computing the exponential function (e^x^) of an assemblage’s Shannon-diversity and dividing that by the assemblage’s OTU richness^24^. This produces a metric that ranges between 0 and 1 with a value of 1 representing complete evenness^24^. As with the other components of alpha diversity, a generalized additive mixed effect model was used to make inferences regarding this metric^22^. The most efficient model included measurement week and capture location as random intercepts. In a separate random effect term, animal ID was included as a random intercept and experimental time was included as a random slope. The gaussian distribution family was indicated for use with this model. Neither a temporal autocorrelation term nor a transformation link was indicated for inclusion in the model.

Since four of seven sham control snakes died during the experiment, we generated generalized additive mixed effects models in order to determine the effect of time on alpha diversity, according to mortality type (natural or euthanasia) within the sham treatment group. More specifically, we generated three models corresponding to each of the measures of alpha diversity examined in this study: OTU richness, Shannon diversity, and Shannon evenness. In each model, a measure of alpha diversity was specified as the response variable. Time and mortality type (natural or euthanasia) were designated as fixed effects. The effect of time was modeled as a smooth term separately for each level of mortality type. Basis complexity (k) was limited to four to prevent model overfitting. Random effects were specified using the ‘bs = re’ argument to the smoothing function. Stepwise AIC selection, as described above, was used to determine inclusion of a random effect, alternative distribution, and/or temporal autocorrelation terms. The most efficient random effects structure for all of these models included measurement week as a random intercept and a separate random effect term where animal ID was specified as a random intercept and experimental time was included as a random slope. For the model of OTU richness, the gamma distribution family with a log transformation link was preferred. For the model of Shannon Diversity, the scaled-t distribution family was preferred. For the model of Shannon evenness, the gaussian distribution family was preferred. A temporal autocorrelation term was not indicated for inclusion in any of these models.

The function *gam.check* from the package *mgcv* was used to ensure that sufficient basis complexity was supplied to all fixed effects terms^22^. Model fit was manually inspected using the function *appraise* from the package *gratia*^25^. Restricted maximum likelihood was used to estimate smoothing parameters by specifying “REML” to the *method* argument. The function *summary* from base R was used to reference model results^16^. The function *predict* from the package *stats* was used to generate model predictions and confidence intervals^16^.

***Analysis of* *β-diversity***

Multidimensional data can be analyzed in terms of both multivariate centroid position, typically representing average assemblage composition, and multivariate dispersion. Multivariate dispersion is a property that describes variation within a group of samples^26^. Given the similarity between this quantitative metric, and ecological theory, multivariate dispersion may be used as a method for quantifying β-diversity^26^. Multivariate dispersion of the host microbiome was assessed to determine the effect of pathogen inoculation on *β*-diversity^26^. We applied the Jaccard, Bray-Curtis, and Raup-Crick dissimilarity indices metric to our dataset using the functions *vegdist* and *raupcrick* from the package *vegan*. The Jaccard index treats compositional data as presence/absence, and therefore, rare species have a greater effect on measured dissimilarity^27^. The Bray-Curtis index accounts for species abundance, and therefore, rare species have less of an effect on measured dissimilarity^27^. The Raup-Crick metric is a presence/absence community dissimilarity metric, which generates a null expectation for the number of shared species between communities by relating global site occupancy of taxa to local site occupancy probabilities, and then accounting for sampling bias likely to occur due to differences in richness between sites^28^. The argument “null=r1” was used to specify that the probability of including a species in a simulated community is proportional to the observed frequency of that species. Using this metric, one is able to explicitly account for differences in richness on measured community dissimilarity^28^. The function *betadisper* in *vegan* was used to generate distance-to-centroid values for each of these community dissimilarity metrics. Communities were grouped by the interaction of experimental time and treatment group. Thus, a multivariate centroid against which distance-to-centroid values can be calculated was created for each treatment group separately at all time points. Distance-to-centroid values were then extracted from the “distances” component of the object created by *betadisper*.

This allowed for linear mixed-effect modeling of distance-to-centroid values through the function *lme* from the package *nlme*^17^. Distance-to-centroid values for each dissimilarity metric were used as the response variable while the fixed effects structure of all three models consisted of treatment group, time, the interaction of these terms, and mortality type. Stepwise AIC selection was used to determine if the inclusion of a random effect, temporal autocorrelation, and/or unequal variance term was appropriate. The selected random effects structure for all three models consisted of animal ID as a random intercept. A temporal autocorrelation term was indicated in all models using the function *corCAR1* from *nlme* with animal ID as an optional grouping variable. An unequal variance term was not included in any of the models. An analysis of variance with type-II sum of squares (Wald χ² statistics; *car* package) was used to determine the significance of the fixed effects. A post-hoc assessment of any significant interaction terms was performed using the *gls* function from *nlme*. For any post-hoc assessments, each treatment group was modeled independently to determine the relationship between multivariate dispersion and time on a per treatment basis. The same fixed effect and temporal autocorrelation terms from the original models were specified in the *gls* models.

***Analysis of* *Assemblage Composition***

The composition of microbial assemblages can be assessed using dissimilarity indices of pairwise comparisons between all of the assemblages within a group^29^. These metrics account for the presence or abundance of OTUs within each assemblage and allow for a multidimensional analysis of assemblage composition^20^. As previously stated, we applied the Jaccard index, the Bray-Curtis index, and the Raup-Crick metric to our dataset. The specified fixed effect terms included treatment group, time, the interaction of these terms, and mortality type. A statistically significant interaction term would indicate average microbiome composition of each treatment group changed in a disparate fashion through time. For all *adonis* models, animal ID was specified as a grouping variable (using “strata=”) to constrain permutations and account for repeated measures.

**REFERENCES**

1. Mebert, K. Good species despite massive hybridization: genetic research on the contact zone between the watersnakes *Nerodia sipedon* and *N. fasciata* in the Carolinas, USA. *Mol. Ecol.* **17**, 1918–1929 (2008).

2. Bohuski, E., Lorch, J. M., Griffin, K. M. & Blehert, D. S. TaqMan real-time polymerase chain reaction for detection of *Ophidiomyces ophiodiicola*, the fungus associated with snake fungal disease. *BMC Vet. Res.* **11**, 95 (2015).

3. Lorch, J. M. *et al.* Experimental infection of snakes with *Ophidiomyces ophiodiicola* causes pathological changes that typify snake fungal disease. *mBio* **6**, e01534-15.

4. Walker, D. M. *et al.* Variability in snake skin microbial assemblages across spatial scales and disease states. *ISME J.* **13**, 2209–2222 (2019).

5. Wiens, J. A. Spatial scaling in ecology. *Funct. Ecol.* **3**, 385–397 (1989).

6. Bohuski, E., Lorch, J. M., Griffin, K. M. & Blehert, D. S. Taqman real-time polymerase chain reaction for detection of ophidiomyces ophiodiicola, the fungus associated with snake fungal disease. *BMC Vet Res* **11**, 95 (2015).

7. Caporaso, J. G. *et al.* Global patterns of 16s rrna diversity at a depth of millions of sequences per sample. *PNAS* **108**, 4516–4522 (2011).

8. Fadrosh, D. W. *et al.* An improved dual-indexing approach for multiplexed 16S rRNA gene sequencing on the Illumina MiSeq platform. *Microbiome* **2**, 6 (2014).

9. Schloss, P. D. *et al.* Introducing mothur: Open-Source, Platform-Independent, Community-Supported Software for Describing and Comparing Microbial Communities. *Appl. Environ. Microbiol.* **75**, 7537–7541 (2009).

10. Kozich, J. J., Westcott, S. L., Baxter, N. T., Highlander, S. K. & Schloss, P. D. Development of a dual-index sequencing strategy and curation pipeline for analyzing amplicon sequence data on the MiSeq Illumina sequencing platform. *Appl Environ Microbiol* **79**, 5112–5120 (2013).

11. Quast, C. *et al.* The SILVA ribosomal RNA gene database project: improved data processing and web-based tools. *Nucleic Acids Res* **41**, D590–D596 (2013).

12. Yilmaz, P. *et al.* The SILVA and “All-species Living Tree Project (LTP)” taxonomic frameworks. *Nucleic Acids Res* **42**, D643–D648 (2014).

13. Gilbert, J. A. & Lynch, S. V. Community ecology as a framework for human microbiome research. *Nature Medicine* **25**, 884–890 (2019).

14. Gotelli, N. J. & Colwell, R. K. Quantifying biodiversity: procedures and pitfalls in the measurement and comparison of species richness. *Ecology Letters* **4**, 379–391 (2001).

15. Weiss, S. *et al.* Normalization and microbial differential abundance strategies depend upon data characteristics. *Microbiome* **5**, 27 (2017).

16. R Core Team. *R: a language and environment for statistical computing*. (R Foundation for Statistical Computing, 2020).

17. Pinheiro, J., Bates, D., DebRoy, S., Sarkar, D. & R Core Team. *Nlme: linear and nonlinear mixed effects models*. (2021).

18. Bozdogan, H. Model selection and akaike’s information criterion (aic): the general theory and its analytical extensions. *Psychometrika* **52**, 345–370 (1987).

19. Fox, J. & Weisberg, S. *An R Companion to Applied Regression*. (SAGE Publications, 2011).

20. Huttenhower, C. *et al.* Structure, function and diversity of the healthy human microbiome. *Nature* **486**, 207–214 (2012).

21. Dixon, P. VEGAN, a package of R functions for community ecology. *Journal of Vegetation Science* **14**, 927–930 (2003).

22. Wood, S. *mgcv: Mixed GAM Computation Vehicle with Automatic Smoothness Estimation*. (2021).

23. Zuur, A., Ieno, E. N., Walker, N., Saveliev, A. A. & Smith, G. M. *Mixed Effects Models and Extensions in Ecology with R*. (Springer-Verlag, 2009). doi:10.1007/978-0-387-87458-6.

24. Heip, C. A new index measuring evenness. *Journal of the Marine Biological Association of the United Kingdom* **54**, 555–557 (1974).

25. Simpson, G. L. & Singmann, H. *Gratia: graceful ’ggplot’-based graphics and other functions for gams fitted using ‘mgcv’*. (2021).

26. Anderson, M. J., Ellingsen, K. E. & McArdle, B. H. Multivariate dispersion as a measure of beta diversity. *Ecology Letters* **9**, 683–693 (2006).

27. Anderson, M. J. *et al.* Navigating the multiple meanings of β diversity: a roadmap for the practicing ecologist. *Ecol. Lett.* **14**, 19–28 (2011).

28. Chase, J. M., Kraft, N. J. B., Smith, K. G., Vellend, M. & Inouye, B. D. Using null models to disentangle variation in community dissimilarity from variation in α-diversity. *Ecosphere* **2**, art24 (2011).

29. Tuomisto, H. A diversity of beta diversities: straightening up a concept gone awry. Part 1. Defining beta diversity as a function of alpha and gamma diversity. *Ecography* **33**, 2–22 (2010).

R code to reproduce analysis below:

# Prepare Working Environment ####

#load required packages

library(tidyverse)

library(installr)

library(rcompanion)

library(ape)

library(ggplot2)

library(gplots)

library(ggord)

library(lme4)

library(glmmTMB)

library(phangorn)

library(plotly)

library(tidymv)

library(tidyr)

library(vegan)

library(tibble)

library(VennDiagram)

library(phyloseq)

library(betapart)

library(car)

library(gridExtra)

library(reshape2)

library(ggsignif)

library(Hmisc)

library(plyr)

library(lmodel2)

library(MuMIn)

library(scales)

library(MASS)

library(nlme)

library(lmerTest)

library(RColorBrewer)

library(ggpubr)

library(gratia)

library(outliers)

library(mgcv)

library(gdata)

library(cowplot)

#override base commands with dplyr commands

filter <- dplyr::filter

select <- dplyr::select

#Set working directory

setwd("G:/My Drive/SFD_MTSU/MS Thesis/Nerodia Project/ANSP R analysis")

options(contrasts = rep("contr.sum", 2)) #set contrasts

theme_set(theme_bw() + # Set plotting theme

theme(panel.grid.major = element_blank(),

panel.grid.minor = element_blank(),

panel.border = element_blank(),

axis.line = element_line(colour = "black"),

axis.text.y = element_text(size=14),

axis.text.x = element_text(size=14),

axis.title.y = element_text(size=14),

axis.title.x = element_text(size=14),

plot.title = element_text(size=30),

legend.position = "bottom",

legend.background = element_rect(fill="white", size=0.5, linetype="solid", colour ="black")))

df <- read.csv("201210.disease.csv", header = TRUE) #import dataframe

#set factors

df$expgroup <- as.factor(df$expgroup)

df$exp.call <- as.factor(df$exp.call)

#set randomized seed

set.seed(1349)

# 1 - Time until mortality is predictive of pathogen load #####

#subset samples from inoculated snakes with Pathogen detection

df.snake.inoc.pos <- df %>%

filter(swab.origin == "snake") %>%

filter(!is.na(exp.time)) %>%

filter(expgroup == "inoculated") %>%

filter(logcopynum != 0)

#Examine structure of response variable

shapiro.test(df.snake.inoc.pos$copy.num) # p-value = 1.905e-15

densityPlot(df.snake.inoc.pos$copy.num, main = "Distribution of Copy Number")

shapiro.test(df.snake.inoc.pos$logcopynum) # p-value = 0.1251

densityPlot(df.snake.inoc.pos$logcopynum, main = "Distribution of Log Copy Number")

#Log transformation makes significant improvement to fit of the data to normal distribution

# Before going through an analysis of log-transformed data, I like to try it with the untransformed data and to take a look at the residuals

# First, let's determine whether the temporal autocorrelation improves model fit

cn1 <- lme(copy.num ~ time.til.death*mortality.type, random = ~ 1 | exp.call , data=df.snake.inoc.pos)

cn2 <- lme(copy.num ~ time.til.death*mortality.type, random = ~ 1 | exp.call , data=df.snake.inoc.pos, correlation = corCAR1(form = ~ exp.time | exp.call))

AIC(cn1, cn2)

# It appears that the temporal autocorrelation does not improve fit and we can proceed with cn1 as the simplest model

# Next, we can add variance structure to cn1 to evaluate whether that improves model fit

# We want to add structures relevant to the explanatory variable in the model, which is a continuous variable

cn3 <- lme(copy.num ~ time.til.death*mortality.type, random = ~ 1 | exp.call , data=df.snake.inoc.pos, weights = varFixed(~time.til.death))

cn4 <- lme(copy.num ~ time.til.death*mortality.type, random = ~ 1 | exp.call , data=df.snake.inoc.pos, weights = varPower(form =~time.til.death))

cn5 <- lme(copy.num ~ time.til.death*mortality.type, random = ~ 1 | exp.call , data=df.snake.inoc.pos, weights = varExp(form =~time.til.death))

cn6 <- lme(copy.num ~ time.til.death*mortality.type, random = ~ 1 | exp.call , data=df.snake.inoc.pos, weights = varFixed(~exp.time))

cn7 <- lme(copy.num ~ time.til.death*mortality.type, random = ~ 1 | exp.call , data=df.snake.inoc.pos, weights = varPower(form =~exp.time))

cn8 <- lme(copy.num ~ time.til.death*mortality.type, random = ~ 1 | exp.call , data=df.snake.inoc.pos, weights = varExp(form =~exp.time))

cn9 <- lme(copy.num ~ time.til.death*mortality.type, random = ~ 1 | exp.call , data=df.snake.inoc.pos, weights = varIdent(form = ~1|mortality.type))

# The models with variances weighted by a fixed and power functions did not converge and do not have AIC values to report

# Therefore, we ask for the AIC values from the values that did converge:

AIC(cn1, cn3, cn4, cn5, cn6, cn7, cn8, cn9)

#Check if using multiple weighted variance structures improves model fit to an appreciable degree

cn10 <- lme(copy.num ~ time.til.death*mortality.type, random = ~ 1 | exp.call , data=df.snake.inoc.pos, weights = varComb(varIdent(form = ~1|mortality.type), varPower(form =~exp.time)))

AIC(cn7, cn9, cn10)

# Then ask for the results of the model

# Again, we can ask for results based on Type II SS to account for imbalance in sample sizes across groups (snakes)

# I'd just adopt asking for Type II SS results throughout the LMM/GLMM/GLM analyses to be consistent, plus the results should not be very different from Type I SS if sample sizes are equal

summary(cn10)

Anova(cn10, type=2)

# Checking the residuals of the best model

E2=resid(cn10, type="normalized")

F2=fitted(cn10)

op=par(mfrow = c(1,2))

MyYlab="Residuals"

plot(x=F2, y=E2, xlab="Fitted Values", ylab=MyYlab)

plot(E2~df.snake.inoc.pos$time.til.death, xlab="time.til.death", ylab=MyYlab)

par(op)

# There are definitely outliers in this analysis, as we would expect

# Let's see what happens when we remove the largest residual outlier

#Rerun this analysis but remove outlier (I'm assuming this is the same outlier you identified above)

df.snake.inoc.pos.removeout <- df.snake.inoc.pos[-c(32),]

cn1.1 <- lme(copy.num ~ time.til.death*mortality.type, random = ~ 1 | exp.call , data=df.snake.inoc.pos.removeout)

cn2.1 <- lme(copy.num ~ time.til.death*mortality.type, random = ~ 1 | exp.call , data=df.snake.inoc.pos.removeout, correlation = corCAR1(form = ~ exp.time | exp.call))

AIC(cn1.1, cn2.1)

cn3.1 <- lme(copy.num ~ time.til.death*mortality.type, random = ~ 1 | exp.call , data=df.snake.inoc.pos.removeout, weights = varFixed(~time.til.death))

cn4.1 <- lme(copy.num ~ time.til.death*mortality.type, random = ~ 1 | exp.call , data=df.snake.inoc.pos.removeout, weights = varPower(form =~time.til.death))

cn5.1 <- lme(copy.num ~ time.til.death*mortality.type, random = ~ 1 | exp.call , data=df.snake.inoc.pos.removeout, weights = varExp(form =~time.til.death))

cn6.1 <- lme(copy.num ~ time.til.death*mortality.type, random = ~ 1 | exp.call , data=df.snake.inoc.pos.removeout, weights = varFixed(~exp.time))

cn7.1 <- lme(copy.num ~ time.til.death*mortality.type, random = ~ 1 | exp.call , data=df.snake.inoc.pos.removeout, weights = varPower(form =~exp.time))

cn8.1 <- lme(copy.num ~ time.til.death*mortality.type, random = ~ 1 | exp.call , data=df.snake.inoc.pos.removeout, weights = varExp(form =~exp.time))

cn9.1 <- lme(copy.num ~ time.til.death*mortality.type, random = ~ 1 | exp.call , data=df.snake.inoc.pos.removeout, weights = varIdent(form = ~1|mortality.type))

AIC(cn1.1, cn3.1, cn4.1, cn5.1, cn6.1, cn7.1, cn8.1, cn9.1)

AIC(cn1.1, cn5.1, cn6.1, cn7.1, cn8.1, cn9.1)

#Check if using multiple weighted variance structures improves model fit to an appreciable degree

cn10.1 <- lme(copy.num ~ time.til.death*mortality.type, random = ~ 1 | exp.call , data=df.snake.inoc.pos.removeout, weights = varComb(varExp(form =~time.til.death), varPower(form =~exp.time)))

cn11.1 <- lme(copy.num ~ time.til.death*mortality.type, random = ~ 1 | exp.call , data=df.snake.inoc.pos.removeout, weights = varComb(varIdent(form = ~1|mortality.type), varExp(form =~time.til.death)))

AIC(cn7.1, cn10.1, cn11.1)

summary(cn10.1)

Anova(cn10.1, type=2)

# Checking the residuals of the best model

E2=resid(cn5.1, type="normalized")

F2=fitted(cn5.1)

op=par(mfrow = c(1,2))

MyYlab="Residuals"

plot(x=F2, y=E2, xlab="Fitted Values", ylab=MyYlab)

plot(E2~df.snake.inoc.pos.removeout$time.til.death, xlab="time.til.death", ylab=MyYlab)

par(op)

# Even with the highest outlier removed, the residuals of the untransformed data indicate that a log-transformation is necessary

# Thus, we can make the decision to move ahead with an analysis of the transformed data, starting with all the data (including the outlier):

cn1.2 <- lme(logcopynum ~ time.til.death*mortality.type, random = ~ 1 | exp.call , data=df.snake.inoc.pos)

cn2.2 <- lme(logcopynum ~ time.til.death*mortality.type, random = ~ 1 | exp.call , data=df.snake.inoc.pos, correlation = corCAR1(form = ~ exp.time | exp.call))

AIC(cn1.2, cn2.2)

cn3.2 <- lme(logcopynum ~ time.til.death*mortality.type, random = ~ 1 | exp.call , data=df.snake.inoc.pos, weights = varFixed(~time.til.death))

cn4.2 <- lme(logcopynum ~ time.til.death*mortality.type, random = ~ 1 | exp.call , data=df.snake.inoc.pos, weights = varPower(form =~time.til.death))

cn5.2 <- lme(logcopynum ~ time.til.death*mortality.type, random = ~ 1 | exp.call , data=df.snake.inoc.pos, weights = varExp(form =~time.til.death))

cn6.2 <- lme(logcopynum ~ time.til.death*mortality.type, random = ~ 1 | exp.call , data=df.snake.inoc.pos, weights = varFixed(~exp.time))

cn7.2 <- lme(logcopynum ~ time.til.death*mortality.type, random = ~ 1 | exp.call , data=df.snake.inoc.pos, weights = varPower(form =~exp.time))

cn8.2 <- lme(logcopynum ~ time.til.death*mortality.type, random = ~ 1 | exp.call , data=df.snake.inoc.pos, weights = varExp(form =~exp.time))

cn9.2 <- lme(logcopynum ~ time.til.death*mortality.type, random = ~ 1 | exp.call , data=df.snake.inoc.pos, weights = varIdent(form = ~1|mortality.type))

AIC(cn1.2, cn3.2, cn4.2, cn5.2, cn6.2, cn7.2, cn8.2, cn9.2)

AIC(cn1.2, cn5.2, cn6.2, cn7.2, cn8.2, cn9.2)

summary(cn6.2)

Anova(cn6.2, type=2)

# Checking the residuals of the best model

E2=resid(cn6.2, type="normalized")

F2=fitted(cn6.2)

op=par(mfrow = c(1,2))

MyYlab="Residuals"

plot(x=F2, y=E2, xlab="Fitted Values", ylab=MyYlab)

plot(E2~df.snake.inoc.pos$time.til.death, xlab="time.til.death", ylab=MyYlab)

par(op)

# post hoc assessment of the trend using a simple gls with the same correlation and variance structure as the best model

cn6.2.post <- gls(logcopynum ~ time.til.death, data=df.snake.inoc.pos)

plot(logcopynum ~ time.til.death, data=df.snake.inoc.pos)

abline(cn6.2.post)

# There is still an outlier when the data are transformed, and we should next examine it's influence on the results

#Rerun this analysis but remove outlier

df.snake.inoc.pos.removeout <- df.snake.inoc.pos[-c(32),]

cn1.3 <- lme(logcopynum ~ time.til.death*mortality.type, random = ~ 1 | exp.call , data=df.snake.inoc.pos.removeout)

cn2.3 <- lme(logcopynum ~ time.til.death*mortality.type, random = ~ 1 | exp.call , data=df.snake.inoc.pos.removeout, correlation = corCAR1(form = ~ exp.time | exp.call))

AIC(cn1.3, cn2.3)

cn3.3 <- lme(logcopynum ~ time.til.death*mortality.type, random = ~ 1 | exp.call , data=df.snake.inoc.pos.removeout, weights = varFixed(~time.til.death))

cn4.3 <- lme(logcopynum ~ time.til.death*mortality.type, random = ~ 1 | exp.call , data=df.snake.inoc.pos.removeout, weights = varPower(form =~time.til.death))

cn5.3 <- lme(logcopynum ~ time.til.death*mortality.type, random = ~ 1 | exp.call , data=df.snake.inoc.pos.removeout, weights = varExp(form =~time.til.death))

cn6.3 <- lme(logcopynum ~ time.til.death*mortality.type, random = ~ 1 | exp.call , data=df.snake.inoc.pos.removeout, weights = varFixed(~exp.time))

cn7.3 <- lme(logcopynum ~ time.til.death*mortality.type, random = ~ 1 | exp.call , data=df.snake.inoc.pos.removeout, weights = varPower(form =~exp.time))

cn8.3 <- lme(logcopynum ~ time.til.death*mortality.type, random = ~ 1 | exp.call , data=df.snake.inoc.pos.removeout, weights = varExp(form =~exp.time))

cn9.3 <- lme(logcopynum ~ time.til.death*mortality.type, random = ~ 1 | exp.call , data=df.snake.inoc.pos.removeout, weights = varIdent(form = ~1|mortality.type))

AIC(cn1.3, cn3.3, cn4.3, cn5.3, cn6.3, cn7.3, cn8.3, cn9.3)

AIC(cn1.3, cn5.3, cn6.3, cn7.3, cn8.3, cn9.3)

summary(cn6.3)

Anova(cn6.3, type=2)

# Checking the residuals of the best model

E2=resid(cn1.3, type="normalized")

F2=fitted(cn1.3)

op=par(mfrow = c(1,2))

MyYlab="Residuals"

plot(x=F2, y=E2, xlab="Fitted Values", ylab=MyYlab)

plot(E2~df.snake.inoc.pos.removeout$time.til.death, xlab="time.til.death", ylab=MyYlab)

par(op)

# post hoc assessment of the trend using a simple gls with the same correlation and variance structure as the best model

cn6.3.post <- gls(logcopynum ~ time.til.death, data=df.snake.inoc.pos.removeout)

plot(logcopynum ~ time.til.death, data=df.snake.inoc.pos.removeout)

abline(cn6.3.post)

# It appears that the results are robust to the inclusion of the outlier - it is a significant relationship either way

# I usually will present the analysis that includes the outlier, unless there is good reason for removing it, such as suspected experimental error

# We can conclude that snakes with higher disease loads died earlier

df.snake.inoc.pos %>%

ggplot(aes(x = time.til.death, y = logcopynum)) +

geom_point() +

geom_smooth(method = 'glm', fill = 'skyblue', color = "black") +

xlab('Days Before Mortality') +

ylab('Pathogen Load') +

annotate(geom = 'text', label = 'p = 0.001562', x = -Inf, y = Inf, hjust = -.2, vjust = 2, size = 6)

# 2 - Time is predictive of pathogen load ####

#subset samples from inoculated snakes with Pathogen detection

df.snake.inoc.pos <- df %>%

filter(swab.origin == "snake") %>%

filter(!is.na(exp.time)) %>%

filter(expgroup == "inoculated") %>%

filter(logcopynum != 0)

# Before going through an analysis of log-transformed data, I like to try it with the untransformed data and to take a look at the residuals

# First, let's determine whether the temporal autocorrelation improves model fit

cn1 <- lme(copy.num ~ exp.time*mortality.type, random = ~ 1 | exp.call , data=df.snake.inoc.pos)

cn2 <- lme(copy.num ~ exp.time*mortality.type, random = ~ 1 | exp.call , data=df.snake.inoc.pos, correlation = corCAR1(form = ~ exp.time | exp.call))

AIC(cn1, cn2)

# It appears that the temporal autocorrelation does not improve fit and we can proceed with cn1 as the simplest model

# Next, we can add variance structure to cn1 to evaluate whether that improves model fit

# We want to add structures relevant to the explanatory variable in the model, which is a continuous variable

cn3 <- lme(copy.num ~ exp.time*mortality.type, random = ~ 1 | exp.call , data=df.snake.inoc.pos, weights = varFixed(~time.til.death))

cn4 <- lme(copy.num ~ exp.time*mortality.type, random = ~ 1 | exp.call , data=df.snake.inoc.pos, weights = varPower(form =~time.til.death))

cn5 <- lme(copy.num ~ exp.time*mortality.type, random = ~ 1 | exp.call , data=df.snake.inoc.pos, weights = varExp(form =~time.til.death))

cn6 <- lme(copy.num ~ exp.time*mortality.type, random = ~ 1 | exp.call , data=df.snake.inoc.pos, weights = varFixed(~exp.time))

cn7 <- lme(copy.num ~ exp.time*mortality.type, random = ~ 1 | exp.call , data=df.snake.inoc.pos, weights = varPower(form =~exp.time))

cn8 <- lme(copy.num ~ exp.time*mortality.type, random = ~ 1 | exp.call , data=df.snake.inoc.pos, weights = varExp(form =~exp.time))

AIC(cn1, cn3, cn4, cn5, cn6, cn7, cn8)

# The models with variances weighted by a fixed and power functions did not converge and do not have AIC values to report

# Therefore, we ask for the AIC values from the values that did converge:

AIC(cn1, cn5, cn6, cn7, cn8)

# Then ask for the results of the model

# Again, we can ask for results based on Type II SS to account for imbalance in sample sizes across groups (snakes)

# I'd just adopt asking for Type II SS results throughout the LMM/GLMM/GLM analyses to be consistent, plus the results should not be very different from Type I SS if sample sizes are equal

summary(cn8)

Anova(cn8, type=2)

# Checking the residuals of the best model

E2=resid(cn8, type="normalized")

F2=fitted(cn8)

op=par(mfrow = c(1,2))

MyYlab="Residuals"

plot(x=F2, y=E2, xlab="Fitted Values", ylab=MyYlab)

plot(E2~df.snake.inoc.pos$time.til.death, xlab="time.til.death", ylab=MyYlab)

par(op)

# There are definitely outliers in this analysis, as we would expect

# Let's see what happens when we remove the largest residual outlier

#Rerun this analysis but remove outlier (I'm assuming this is the same outlier you identified above)

df.snake.inoc.pos.removeout <- df.snake.inoc.pos[-c(32),]

cn1.1 <- lme(copy.num ~ exp.time*mortality.type, random = ~ 1 | exp.call , data=df.snake.inoc.pos.removeout)

cn2.1 <- lme(copy.num ~ exp.time*mortality.type, random = ~ 1 | exp.call , data=df.snake.inoc.pos.removeout, correlation = corCAR1(form = ~ exp.time | exp.call))

AIC(cn1.1, cn2.1)

cn3.1 <- lme(copy.num ~ exp.time*mortality.type, random = ~ 1 | exp.call , data=df.snake.inoc.pos.removeout, weights = varFixed(~time.til.death))

cn4.1 <- lme(copy.num ~ exp.time*mortality.type, random = ~ 1 | exp.call , data=df.snake.inoc.pos.removeout, weights = varPower(form =~time.til.death))

cn5.1 <- lme(copy.num ~ exp.time*mortality.type, random = ~ 1 | exp.call , data=df.snake.inoc.pos.removeout, weights = varExp(form =~time.til.death))

cn6.1 <- lme(copy.num ~ exp.time*mortality.type, random = ~ 1 | exp.call , data=df.snake.inoc.pos.removeout, weights = varFixed(~exp.time))

cn7.1 <- lme(copy.num ~ exp.time*mortality.type, random = ~ 1 | exp.call , data=df.snake.inoc.pos.removeout, weights = varPower(form =~exp.time))

cn8.1 <- lme(copy.num ~ exp.time*mortality.type, random = ~ 1 | exp.call , data=df.snake.inoc.pos.removeout, weights = varExp(form =~exp.time))

AIC(cn1.1, cn3.1, cn4.1, cn5.1, cn6.1, cn7.1, cn8.1)

AIC(cn1.1, cn5.1, cn6.1, cn7.1, cn8.1)

summary(cn7.1)

Anova(cn7.1, type=2)

# Checking the residuals of the best model

E2=resid(cn5.1, type="normalized")

F2=fitted(cn5.1)

op=par(mfrow = c(1,2))

MyYlab="Residuals"

plot(x=F2, y=E2, xlab="Fitted Values", ylab=MyYlab)

plot(E2~df.snake.inoc.pos.removeout$time.til.death, xlab="time.til.death", ylab=MyYlab)

par(op)

# Even with the highest outlier removed, the residuals of the untransformed data indicate that a log-transformation is necessary

# Thus, we can make the decision to move ahead with an analysis of the transformed data, starting with all the data (including the outlier):

cn1.2 <- lme(logcopynum ~ exp.time*mortality.type, random = ~ 1 | exp.call , data=df.snake.inoc.pos)

cn2.2 <- lme(logcopynum ~ exp.time*mortality.type, random = ~ 1 | exp.call , data=df.snake.inoc.pos, correlation = corCAR1(form = ~ exp.time | exp.call))

AIC(cn1.2, cn2.2)

cn3.2 <- lme(logcopynum ~ exp.time*mortality.type, random = ~ 1 | exp.call , data=df.snake.inoc.pos, weights = varFixed(~time.til.death))

cn4.2 <- lme(logcopynum ~ exp.time*mortality.type, random = ~ 1 | exp.call , data=df.snake.inoc.pos, weights = varPower(form =~time.til.death))

cn5.2 <- lme(logcopynum ~ exp.time*mortality.type, random = ~ 1 | exp.call , data=df.snake.inoc.pos, weights = varExp(form =~time.til.death))

cn6.2 <- lme(logcopynum ~ exp.time*mortality.type, random = ~ 1 | exp.call , data=df.snake.inoc.pos, weights = varFixed(~exp.time))

cn7.2 <- lme(logcopynum ~ exp.time*mortality.type, random = ~ 1 | exp.call , data=df.snake.inoc.pos, weights = varPower(form =~exp.time))

cn8.2 <- lme(logcopynum ~ exp.time*mortality.type, random = ~ 1 | exp.call , data=df.snake.inoc.pos, weights = varExp(form =~exp.time))

AIC(cn1.2, cn3.2, cn4.2, cn5.2, cn6.2, cn7.2, cn8.2)

AIC(cn1.2, cn5.2, cn6.2, cn7.2, cn8.2)

summary(cn6.2)

Anova(cn6.2, type=2)

# Checking the residuals of the best model

E2=resid(cn6.2, type="normalized")

F2=fitted(cn6.2)

op=par(mfrow = c(1,2))

MyYlab="Residuals"

plot(x=F2, y=E2, xlab="Fitted Values", ylab=MyYlab)

plot(E2~df.snake.inoc.pos$time.til.death, xlab="time.til.death", ylab=MyYlab)

par(op)

# post hoc assessment of the trend using a simple gls with the same correlation and variance structure as the best model

cn6.2.post <- gls(logcopynum ~ exp.time, data=df.snake.inoc.pos)

plot(logcopynum ~ exp.time, data=df.snake.inoc.pos)

abline(cn6.2.post)

# There is still an outlier when the data are transformed, and we should next examine it's influence on the results

#Rerun this analysis but remove outlier

df.snake.inoc.pos.removeout <- df.snake.inoc.pos[-c(32),]

cn1.3 <- lme(logcopynum ~ exp.time*mortality.type, random = ~ 1 | exp.call , data=df.snake.inoc.pos.removeout)

cn2.3 <- lme(logcopynum ~ exp.time*mortality.type, random = ~ 1 | exp.call , data=df.snake.inoc.pos.removeout, correlation = corCAR1(form = ~ exp.time | exp.call))

AIC(cn1.3, cn2.3)

cn3.3 <- lme(logcopynum ~ exp.time*mortality.type, random = ~ 1 | exp.call , data=df.snake.inoc.pos.removeout, weights = varFixed(~time.til.death))

cn4.3 <- lme(logcopynum ~ exp.time*mortality.type, random = ~ 1 | exp.call , data=df.snake.inoc.pos.removeout, weights = varPower(form =~time.til.death))

cn5.3 <- lme(logcopynum ~ exp.time*mortality.type, random = ~ 1 | exp.call , data=df.snake.inoc.pos.removeout, weights = varExp(form =~time.til.death))

cn6.3 <- lme(logcopynum ~ exp.time*mortality.type, random = ~ 1 | exp.call , data=df.snake.inoc.pos.removeout, weights = varFixed(~exp.time))

cn7.3 <- lme(logcopynum ~ exp.time*mortality.type, random = ~ 1 | exp.call , data=df.snake.inoc.pos.removeout, weights = varPower(form =~exp.time))

cn8.3 <- lme(logcopynum ~ exp.time*mortality.type, random = ~ 1 | exp.call , data=df.snake.inoc.pos.removeout, weights = varExp(form =~exp.time))

AIC(cn1.3, cn3.3, cn4.3, cn5.3, cn6.3, cn7.3, cn8.3)

AIC(cn1.3, cn5.3, cn6.3, cn7.3, cn8.3)

summary(cn6.3)

Anova(cn6.3, type=2)

# Checking the residuals of the best model

E2=resid(cn1.3, type="normalized")

F2=fitted(cn1.3)

op=par(mfrow = c(1,2))

MyYlab="Residuals"

plot(x=F2, y=E2, xlab="Fitted Values", ylab=MyYlab)

plot(E2~df.snake.inoc.pos.removeout$time.til.death, xlab="time.til.death", ylab=MyYlab)

par(op)

# post hoc assessment of the trend using a simple gls with the same correlation and variance structure as the best model

cn6.3.post <- gls(logcopynum ~ exp.time, data=df.snake.inoc.pos.removeout)

plot(logcopynum ~ exp.time, data=df.snake.inoc.pos.removeout)

abline(cn6.3.post)

# It appears that the results are robust to the inclusion of the outlier - it is a significant relationship either way

# I usually will present the analysis that includes the outlier, unless there is good reason for removing it, such as suspected experimental error

# We can conclude that snakes that animals had higher disease loads later in the trial

cn.fig1<-df.snake.inoc.pos %>%

ggplot(aes(x = exp.time, y = logcopynum)) +

geom_point(size = 2, alpha = 0.5) +

geom_smooth(method = 'glm', fill = 'skyblue', color = "black") +

xlab('Time (days)') +

ylab('Pathogen load') +

ggtitle("b") +

annotate(geom = 'text', label = 'p = 0.003', x = -Inf, y = Inf, hjust = -.2, vjust = 2, size = 5)

cn.fig2<-df.snake.inoc.pos %>%

ggplot(aes(x = time.til.death, y = logcopynum)) +

geom_point(size = 2, alpha = 0.5) +

geom_smooth(method = 'glm', fill = 'skyblue', color = "black") +

xlab('Days before mortality') +

ylab('Pathogen load') +

ggtitle("a") +

annotate(geom = 'text', label = 'p = 0.002', x = -Inf, y = Inf, hjust = -.2, vjust = 2, size = 5)

fig1 <- ggarrange(cn.fig2, cn.fig1)

fig1

# 3 - Time by treatment is predictive of host microbiome richness ####

#Set up dataframe of samples taken via weekly swabbing regime

df.snake <- df %>%

filter(swab.origin == "snake") %>%

filter(!is.na(exp.time))

df.snake$week <- factor(df.snake$week, levels = c("w1", "w2", "w3", "w4", "w5", "w6", "w7", "w8", "w9", "w10", "w11", "w12", "w13"))

df.snake$cap.location <- as.factor(df.snake$cap.location)

df.snake$mortality.type <- as.factor(df.snake$mortality.type)

snake.otu <- df.snake[,23:6880]

#Examine structure of response variable

shapiro.test(df.snake$richness)

plotNormalDensity(df.snake$richness, main = 'distribution of richness')

shapiro.test(log(df.snake$richness))

plotNormalDensity(log(df.snake$richness), main = 'distribution of log richness')

# Log transformation makes minor improvements to fit of the data

# GAMM model

# first, evaluate whether to specify random intercepts or random intercepts and slopes

rich.1 <- gam(richness ~ s(exp.time, by = expgroup, k = 4) + expgroup + mortality.type + s(week, bs = "re") + s(exp.call, exp.time, bs = 're') + s(expgroup, bs = "re") + s(cap.location, bs = "re"), family = gaussian(link = "identity"), data=df.snake, select = TRUE, method = "REML")

rich.2 <- gam(richness ~ s(exp.time, by = expgroup, k = 4) + expgroup + mortality.type + s(week, bs = "re") + s(exp.call, exp.time, bs = 're'), family = gaussian(link = "identity"), data=df.snake, select = TRUE, method = "REML")

AIC(rich.1,rich.2)

#next assess whether to include an alternative distribution function or transformation link

rich.3 <- gam(richness ~ s(exp.time, by = expgroup, k = 4) + expgroup + mortality.type + s(week, bs = "re")+ s(exp.call, exp.time, bs = 're'),family = gaussian(link = "log"), data=df.snake, method = "REML")

rich.4 <- gam(richness ~ s(exp.time, by = expgroup, k = 4) + expgroup + mortality.type + s(week, bs = "re")+ s(exp.call, exp.time, bs = 're'),family = Gamma(link = "identity"), data=df.snake, method = "REML")

rich.5 <- gam(richness ~ s(exp.time, by = expgroup, k = 4) + expgroup + mortality.type + s(week, bs = "re")+ s(exp.call, exp.time, bs = 're'),family = Gamma(link = "log"), data=df.snake, method = "REML")

rich.6 <- gam(richness ~ s(exp.time, by = expgroup, k = 4) + expgroup + mortality.type + s(week, bs = "re")+ s(exp.call, exp.time, bs = 're'),family = mgcv::scat(link = "identity"), data=df.snake, method = "REML")

rich.7 <- gam(richness ~ s(exp.time, by = expgroup, k = 4) + expgroup + mortality.type + s(week, bs = "re")+ s(exp.call, exp.time, bs = 're'),family = mgcv::scat(link = "log"), data=df.snake, method = "REML")

rich.8 <- gam(richness ~ s(exp.time, by = expgroup, k = 4) + expgroup + mortality.type + s(week, bs = "re")+ s(exp.call, exp.time, bs = 're'),family = poisson(link = "identity"), data=df.snake, method = "REML")

rich.9 <- gam(richness ~ s(exp.time, by = expgroup, k = 4) + expgroup + mortality.type + s(week, bs = "re")+ s(exp.call, exp.time, bs = 're'),family = poisson(link = "log"), data=df.snake, method = "REML")

AIC(rich.2,rich.3,rich.4,rich.5,rich.6,rich.7,rich.8,rich.9) #rich.4 preferred

# next assess whether to include a temporal autocorrelation term

rich.11 <- gam(richness ~ s(exp.time, by = expgroup, k = 4) + expgroup + mortality.type + s(week, bs = "re")+ s(exp.call, exp.time, bs = 're'),family = Gamma(link = "identity"), data=df.snake,correlation = corCAR1(form = ~ exp.time | exp.call), method = "REML")

AIC(rich.4,rich.11)

summary(rich.4)

k.check(rich.4)

appraise(rich.4, method = "simulate", n_simulate = 1000) # Examine model fit

draw(rich.4)

df.snake$predict <- predict(rich.4, exclude = c("s(week)", "s(exp.call,exp.time)"))

rich.inoc <- subset(df.snake, expgroup == "inoculated") %>%

ggplot(aes(x=exp.time, y=richness)) +

geom_point(color = "#E41A1C", size = 2, alpha = 0.75) +

geom_smooth(aes(y = predict), color = "#E41A1C", se = T) +

scale_y_continuous(limits = c(0,250), breaks = c(50, 100, 150, 200, 250)) +

scale_x_continuous(limits = c(0,80), breaks = seq(0, 80, by = 10)) +

xlab("Time (days)") +

ylab("OTU Richness") +

ggtitle("a") +

annotate(geom = 'text', label = 'p = 0.013', x = -Inf, y = Inf, hjust = -2, vjust = 1, size = 5)

rich.sham <- subset(df.snake, expgroup == "sham") %>%

ggplot(aes(x=exp.time, y=richness)) +

geom_point(color = "#377EB8", size = 2, alpha = 0.75, shape = "triangle") +

geom_hline(yintercept = mean(subset(df.snake, expgroup == "sham")$richness), color = "#377EB8", lty = "longdash", size = 1) +

scale_y_continuous(limits = c(0,250), breaks = c(50, 100, 150, 200, 250)) +

scale_x_continuous(limits = c(0,80), breaks = seq(0, 80, by = 10)) +

xlab("Time (days)") +

ylab("OTU Richness") +

ggtitle("b") +

annotate(geom = 'text', label = 'p = 0.117', x = -Inf, y = Inf, hjust = -2, vjust = 1, size = 5)

ggarrange(rich.inoc, rich.sham)

# 4 - Time by treatment is predictive of host microbiome alpha diversity #####

#Set up dataframe of samples taken via weekly swabbing regime

df.snake <- df %>%

filter(swab.origin == "snake") %>%

filter(!is.na(exp.time))

df.snake$week <- factor(df.snake$week, levels = c("w1", "w2", "w3", "w4", "w5", "w6", "w7", "w8", "w9", "w10", "w11", "w12", "w13"))

df.snake$cap.location <- as.factor(df.snake$cap.location)

df.snake$mortality.type <- as.factor(df.snake$mortality.type)

snake.otu <- df.snake[,23:6880]

#Examine structure of response variable

shapiro.test(df.snake$shannon) # p-value = 3.059e-07

densityplot(df.snake$shannon, main = 'distribution of shannon diversity')

shapiro.test(log(df.snake$shannon)) # p-value = 8.982e-15

densityplot(log(df.snake$shannon), main = 'distribution of log shannon diversity')

# Log transformation appears to worsen the fit of the data to the normal distribution

# GAMM model of shannon diversity values

# first, evaluate whether to specify random intercepts or random intercepts and slopes

shan.1 <- gam(shannon ~ s(exp.time, by = expgroup, k = 4) + expgroup + mortality.type + s(week, bs = "re") + s(exp.call, exp.time, bs = 're') + s(expgroup, bs = "re"),family = gaussian(link = "identity"), data=df.snake, method = "REML")

shan.2 <- gam(shannon ~ s(exp.time, by = expgroup, k = 4) + expgroup + mortality.type + s(week, bs = "re") + s(exp.call, exp.time, bs = 're'), family = gaussian(link = "identity"), data=df.snake, method = "REML")

shan.3 <- gam(shannon ~ s(exp.time, by = expgroup, k = 4) + expgroup + mortality.type + s(exp.call, exp.time, bs = 're'), family = gaussian(link = "identity"), data=df.snake, method = "REML")

AIC(shan.1, shan.2, shan.3)

summary(shan.3)

#next assess whether to include an alternative distribution function or transformation link

shan.4 <- gam(shannon ~ s(exp.time, by = expgroup, k = 4) + expgroup + mortality.type + s(exp.call, exp.time, bs = 're'), family = gaussian(link = "log"), data=df.snake, method = "REML")

shan.5 <- gam(shannon ~ s(exp.time, by = expgroup, k = 4) + expgroup + mortality.type + s(exp.call, exp.time, bs = 're'), family = Gamma(link = "identity"), data=df.snake, method = "REML")

shan.6 <- gam(shannon ~ s(exp.time, by = expgroup, k = 4) + expgroup + mortality.type + s(exp.call, exp.time, bs = 're'), family = Gamma(link = "log"), data=df.snake, method = "REML")

shan.7 <- gam(shannon ~ s(exp.time, by = expgroup, k = 4) + expgroup + mortality.type + s(exp.call, exp.time, bs = 're'), family = mgcv::scat(link = "identity"), data=df.snake, method = "REML")

shan.8 <- gam(shannon ~ s(exp.time, by = expgroup, k = 4) + expgroup + mortality.type + s(exp.call, exp.time, bs = 're'), family = mgcv::scat(link = "log"), data=df.snake, method = "REML")

AIC(shan.3,shan.4,shan.5,shan.6, shan.7, shan.8) #shan.7 preferred

# next assess whether to include a temporal autocorrelation term

shan.11 <- gam(shannon ~ s(exp.time, by = expgroup, k = 4) + expgroup + mortality.type + s(exp.call, exp.time, bs = 're'), family = mgcv::scat(link = "identity"), data=df.snake, correlation = corCAR1(form = ~ exp.time | exp.call), method = "REML")

AIC(shan.7,shan.11)

# Examine diagnostic plots of model

k.check(shan.7)

appraise(shan.7, method = "simulate", n_simulate = 1000)

draw(shan.7)

summary(shan.7) # Note that trend is significant only for inoculated animals under GAM model

df.snake$predict <- predict(shan.7, exclude = "s(exp.call,exp.time)")

shan.inoc <- subset(df.snake, expgroup == "inoculated") %>%

ggplot(aes(x=exp.time, y=shannon)) +

geom_point(color = "#E41A1C", size = 2, alpha = 0.75) +

geom_smooth(aes(y = predict), color = "#E41A1C", se = T) +

scale_y_continuous(limits = c(0,5), breaks = c(1, 2, 3, 4, 5)) +

scale_x_continuous(limits = c(0,80), breaks = seq(0, 80, by = 10)) +

xlab("Time (days)") +

ylab("Shannon Diversity") +

ggtitle("c") +

annotate(geom = 'text', label = 'p < 0.001', x = -Inf, y = Inf, hjust = -1.5, vjust = 1, size = 5)

shan.sham <- subset(df.snake, expgroup == "sham") %>%

ggplot(aes(x=exp.time, y=shannon)) +

geom_point(color = "#377EB8", size = 2, alpha = 0.75, shape = "triangle") +

geom_hline(yintercept = mean(subset(df.snake, expgroup == "sham")$shannon), color = "#377EB8", lty = "longdash", size = 1) +

scale_y_continuous(limits = c(0,5), breaks = c(1, 2, 3, 4, 5)) +

scale_x_continuous(limits = c(0,80), breaks = seq(0, 80, by = 10)) +

xlab("Time (days)") +

ylab("Shannon Diversity") +

ggtitle("d") +

annotate(geom = 'text', label = 'p = 0.217', x = -Inf, y = Inf, hjust = -1.5, vjust = 1, size = 5)

legend <- get_legend(

df.snake %>%

mutate(expgroup = revalue(expgroup, c("inoculated" = "Inoculated", 'sham' = 'Sham'))) %>%

ggplot(aes(x = exp.time, y = shannon, color = expgroup, shape = expgroup)) +

geom_point(size = 2, alpha = 0.75) +

scale_color_brewer(palette = "Set1") +

labs(color = "Treatment",

shape = "Treatment")

)

prow <- plot_grid(rich.inoc, rich.sham, shan.inoc, shan.sham, align = 'v')

fig2 <- plot_grid(prow,legend, nrow = 2, rel_heights = c(3,0.4))

fig2

# 5 - Time by treatment is predictive of host microbiome evenness #####

#Set up dataframe of samples taken via weekly swabbing regime

df.snake <- df %>%

filter(swab.origin == "snake") %>%

filter(!is.na(exp.time))

df.snake$week <- factor(df.snake$week, levels = c("w1", "w2", "w3", "w4", "w5", "w6", "w7", "w8", "w9", "w10", "w11", "w12", "w13"))

df.snake$cap.location <- as.factor(df.snake$cap.location)

df.snake$mortality.type <- as.factor(df.snake$mortality.type)

snake.otu <- df.snake[,23:6880]

# calculate shannon evenness

df.snake$evenness <- exp(df.snake$shannon)/(df.snake$richness)

# Exploratory visualization

df.snake %>%

ggplot(aes(x = exp.time, y = evenness, color = expgroup)) +

facet_wrap(~expgroup) +

geom_point(alpha = 0.2, aes(shape = expgroup), size = 2.5) +

geom_smooth(method = "gam", lty = 2, alpha = 0.5) +

scale_color_brewer(palette = "Set1")

#Examine structure of response variable

shapiro.test(df.snake$evenness) # p-value = 0.1543

plotNormalDensity(df.snake$evenness, main = "Distribution of Copy Number")

#data is normally distributed with heavy tails

#Check for outliers

grubbs.test(df.snake$evenness)

boxplot(df.snake$evenness)

#no outliers

# GAMM model of shannon diversity values

# first, evaluate whether to specify random intercepts or random intercepts and slopes

even.1 <- gam(evenness ~ s(exp.time, by = expgroup, k = 4) + expgroup + mortality.type + s(week, bs = "re") + s(exp.call, exp.time, bs = 're') + s(expgroup, bs = "re"),family = gaussian(link = "identity"), data=df.snake, method = "REML")

even.2 <- gam(evenness ~ s(exp.time, by = expgroup, k = 4) + expgroup + mortality.type + s(week, bs = "re") + s(exp.call, exp.time, bs = 're'),family = gaussian(link = "identity"), data=df.snake, method = "REML")

AIC(even.1, even.2)

#next assess whether to include an alternative distribution function or transformation link

even.3 <- gam(evenness ~ s(exp.time, by = expgroup, k = 4) + expgroup + mortality.type + s(week, bs = "re") + s(exp.call, exp.time, bs = 're'),family = gaussian(link = "log"), data=df.snake, method = "REML")

even.4 <- gam(evenness ~ s(exp.time, by = expgroup, k = 4) + expgroup + mortality.type + s(week, bs = "re") + s(exp.call, exp.time, bs = 're'),family = mgcv::scat(link = "identity"), data=df.snake, method = "REML")

even.4 <- gam(evenness ~ s(exp.time, by = expgroup, k = 4) + expgroup + mortality.type + s(week, bs = "re") + s(exp.call, exp.time, bs = 're'),family = mgcv::scat(link = "log"), data=df.snake, method = "REML")

AIC(even.2,even.3, even.4) #normal distribution most efficient which makes sense given shapiro-test result

# next assess whether to include a temporal autocorrelation term

even.5 <- gam(evenness ~ s(exp.time, by = expgroup, k = 4) + expgroup + mortality.type + s(week, bs = "re") + s(exp.call, exp.time, bs = 're'),family = gaussian(link = "identity"), correlation = corCAR1(form = ~ exp.time | exp.call), data=df.snake, method = "REML")

AIC(even.2,even.5)

# Examine diagnostic plots of model

k.check(even.2)

appraise(even.2, method = 'simulate', n_simulate = 1000)

summary(even.2)

draw(even.2)

df.snake$predict <- predict(even.2, se.fit = F, exclude = c("s(week)", "s(exp.call,exp.time)"))

even.inoc <- subset(df.snake, expgroup == "inoculated") %>%

ggplot(aes(x=exp.time, y=evenness)) +

geom_point(color = "#E41A1C", size = 2, alpha = 0.75) +

geom_smooth(aes(y = predict), color = "#E41A1C", se = T) +

scale_x_continuous(limits = c(0,80), breaks = seq(0, 80, by = 10)) +

scale_y_continuous(limits = c(0,1)) +

xlab("Time (days)") +

ylab("Shannon Evenness") +

ggtitle("e") +

annotate(geom = 'text', label = 'p = 0.043', x = -Inf, y = Inf, hjust = -2.5, vjust = 1, size = 5)

even.sham <- subset(df.snake, expgroup == "sham") %>%

ggplot(aes(x=exp.time, y=evenness)) +

geom_point(color = "#377EB8", size = 2, alpha = 0.75, shape = "triangle") +

geom_hline(yintercept = mean(subset(df.snake, expgroup == "sham")$evenness), color = "#377EB8", lty = "longdash", size = 1) +

scale_x_continuous(limits = c(0,80), breaks = seq(0, 80, by = 10)) +

scale_y_continuous(limits = c(0,1)) +

xlab("Time (days)") +

ylab("Shannon Evenness") +

ggtitle("f") +

annotate(geom = 'text', label = 'p = 0.967', x = -Inf, y = Inf, hjust = -2, vjust = 1, size = 5)

p <- plot_grid(rich.inoc, rich.sham, shan.inoc, shan.sham, even.inoc, even.sham, ncol = 2, nrow = 3, align = 'v')

fig2 <- plot_grid(p,legend, nrow = 2, rel_heights = c(4.5,0.4))

fig2

# 6 - Richness & disease interact to predict host microbiome composition ####

mycontourcolors <- c("#FFFFFF",brewer.pal(n = 9, name = 'Greys')[2:9]) #set color scale for plotting contours

mypointcolors <- c(brewer.pal(n=3, name = 'Set1')[1:2])

#Set up dataframe of samples taken via weekly swabbing regime

df.snake <- df %>%

filter(swab.origin == "snake") %>%

filter(!is.na(exp.time))

df.snake$week <- factor(df.snake$week, levels = c("w1", "w2", "w3", "w4", "w5", "w6", "w7", "w8", "w9", "w10", "w11", "w12", "w13"))

df.snake <- mutate(df.snake, expgroup = revalue(expgroup, c("inoculated" = "Inoculated", 'sham' = 'Sham')))

snake.otu <- df.snake[,23:6880]

# Jaccard

# Analysis of Multivariate centroid position (community structure)

jaccard.snake.otu <- vegdist(snake.otu, method = "jaccard", na.rm = TRUE) # Generate Jaccard dissimilarity matrix, This metric is P/A and excludes joint absences

#PERMANOVA model of community composition data

adonis(jaccard.snake.otu ~ expgroup*exp.time + mortality.type,

data = df.snake,

permutations = 999,

method = "jaccard",

strata = df.snake$exp.call) # PERMANOVA using Jaccard dissimilarity

# Perform Nonmetric Multidimensional Scaling and extract points

mds1 <- metaMDS(jaccard.snake.otu, k=5, try = 50, trace = 0) # stress = 0.1273001

mds1_data <- as.data.frame(mds1$points)

df.snake$MDS1 <- mds1_data$MDS1

df.snake$MDS2 <- mds1_data$MDS2

# Significant differences between sham / inoculated snake microbiome structure

snake.structure.fig1 <- ggplot(df.snake, aes(x = MDS1, y = MDS2, z = exp.time)) +

stat_density_2d_filled(aes(fill = ..level..), contour = T, bins = 9, show.legend = F) +

geom_point(aes(shape=expgroup, color = expgroup), size = 2.5) +

stat_ellipse(size = 1, aes(color = expgroup)) +

labs(title = 'a',

x = "MDS1",

y = "MDS2",

color = "Treatment",

shape = "Treatment") +

scale_colour_manual(values = mypointcolors) +

scale_fill_manual(values = mycontourcolors) +

annotate(geom = 'text', label = 'p = 0.020', x = -Inf, y = Inf, hjust = -.1, vjust = 1.2, size = 5) +

theme(legend.position = "none")

# Bray-Curtis

# Analysis of Multivariate centroid position (community structure)

bray.snake.otu <- vegdist(snake.otu, method = "bray", na.rm = TRUE) # Generate Jaccard dissimilarity matrix, This metric is P/A and excludes joint absences

#PERMANOVA model of community composition data

adonis(bray.snake.otu ~ expgroup*exp.time + mortality.type,

data = df.snake,

permutations = 999,

strata = df.snake$exp.call) # PERMANOVA using Jaccard dissimilarity

# Perform Nonmetric Multidimensional Scaling and extract points

mds1 <- metaMDS(bray.snake.otu, k=5, try = 50, trace = 0) # stress = 0.1234844

mds1_data <- as.data.frame(mds1$points)

df.snake$MDS3 <- mds1_data$MDS1

df.snake$MDS4 <- mds1_data$MDS2

# Significant differences between sham / inoculated snake microbiome structure

snake.structure.fig2 <- ggplot(df.snake, aes(x = MDS3, y = MDS4, z = exp.time)) +

stat_density_2d_filled(aes(fill = ..level..), contour = T, bins = 9, show.legend = F) +

geom_point(aes(shape=expgroup, color = expgroup), size = 2.5) +

stat_ellipse(size = 1, aes(color = expgroup)) +

labs(title = 'b',

x = "MDS1",

y = "MDS2",

color = "Treatment",

shape = "Treatment") +

scale_colour_manual(values = mypointcolors) +

scale_fill_manual(values = mycontourcolors) +

annotate(geom = 'text', label = 'p = 0.038', x = -Inf, y = Inf, hjust = -.1, vjust = 1.2, size = 5) +

theme(legend.position = "none")

# Raup-crick

#raup.snake.otu <- raupcrick(snake.otu, null = 'r1', nsimul = 999)

#save(raup.snake.otu, file = 'raup.snake.otu.rda')

load('raup.snake.otu.rda')

#PERMANOVA model of community composition data

adonis(raup.snake.otu ~ expgroup*exp.time + mortality.type, #no significant interaction term when specifically accounting for differences in richness

data = df.snake,

permutations = 999,

strata = df.snake$exp.call)

# Perform Nonmetric Multidimensional Scaling and extract points

mds1 <- metaMDS(raup.snake.otu, k=5, try = 50, trace = 0) # stress = 0.1089634

mds1_data <- as.data.frame(mds1$points)

df.snake$MDS5 <- mds1_data$MDS1

df.snake$MDS6 <- mds1_data$MDS2

# Significant differences between experimental groups when accounting for richness explicitly (raup-crick)

snake.structure.fig3 <- ggplot(df.snake, aes(x = MDS5, y = MDS6, z = exp.time)) +

stat_density_2d_filled(aes(fill = ..level..), contour = T, bins = 9, show.legend = F) +

geom_point(aes(shape=expgroup, color = expgroup), size = 2.5) +

stat_ellipse(size = 1, aes(color = expgroup)) +

labs(title = 'c',

x = "MDS1",

y = "MDS2",

color = "Treatment",

shape = "Treatment") +

scale_colour_manual(values = mypointcolors) +

scale_fill_manual(values = mycontourcolors) +

annotate(geom = 'text', label = 'p = 0.307', x = -Inf, y = Inf, hjust = -.1, vjust = 1.2, size = 5) +

theme(legend.position = "none")

# Generate multipanel figure that summarizes results of this section

legend <- get_legend(ggplot(df.snake, aes(x = MDS5, y = MDS6, z = exp.time)) + stat_density_2d_filled(aes(fill = ..level..), contour = T, bins = 9, show.legend = F) + geom_point(aes(shape=expgroup, color = expgroup), size = 2.5) + stat_ellipse(size = 1, aes(color = expgroup)) + theme_classic() + theme(legend.background = element_rect(fill="white", size=0.5, linetype="solid", colour ="black"), legend.position = "bottom") + labs(title = 'c', x = "MDS1", y = "MDS2", color = "Treatment", shape = "Treatment") + scale_colour_manual(values = mypointcolors) + scale_fill_manual(values = mycontourcolors) + annotate(geom = 'text', label = 'p = 0.346', x = -Inf, y = Inf, hjust = -.1, vjust = 1.2, size = 5))

grid.arrange(snake.structure.fig1, snake.structure.fig2, snake.structure.fig3,

legend, ncol=3, nrow = 2,

layout_matrix = rbind(c(1,2,3), c(4,4,4)),

widths = c(2.7, 2.7, 2.7), heights = c(2.5, 0.2))

# 7 - Higher B-diversity in inoculated host microbiome #####

#Set up dataframe of samples taken via weekly swabbing regime

df.snake <- df %>%

filter(swab.origin == "snake") %>%

filter(!is.na(exp.time))

df.snake$week <- factor(df.snake$week, levels = c("w1", "w2", "w3", "w4", "w5", "w6", "w7", "w8", "w9", "w10", "w11", "w12", "w13"))

df.snake$cap.location <- as.factor(df.snake$cap.location)

df.snake$mortality.type <- as.factor(df.snake$mortality.type)

snake.otu <- df.snake[,23:6880]

# Generate/Load multivariate dissimilarity matrices

jaccard.snake.otu <- vegdist(snake.otu, method = "jaccard", na.rm = TRUE) # Generate Jaccard dissimilarity matrix

bray.snake.otu <- vegdist(snake.otu, method = "bray", na.rm = TRUE) # Generate Bray-Cutis dissimilarity matrix

load('raup.snake.otu.rda') # Load Raup-crick dissimilarity matrix

# Calculate distance to centroid values

jacc.snake <- betadisper(jaccard.snake.otu, group = interaction(df.snake$expgroup, df.snake$exp.time), type= c("centroid"), bias.adjust=FALSE, add=FALSE)

bray.snake <- betadisper(bray.snake.otu, group = interaction(df.snake$expgroup, df.snake$exp.time), type= c("centroid"), bias.adjust=FALSE, add=FALSE)

raup.snake <- betadisper(raup.snake.otu, group = interaction(df.snake$expgroup, df.snake$exp.time), type= c("centroid"), bias.adjust=FALSE, add=FALSE)

# Create dataframe containing distance to centroid values for analysis

df.disper.snake <- data.frame(jacc = jacc.snake$distances,

bray = bray.snake$distances,

raup = raup.snake$distances,

expgroup = df.snake$expgroup,

week = df.snake$week,

exp.time = as.numeric(df.snake$exp.time),

exp.call = df.snake$exp.call,

cap.location = df.snake$cap.location,

mortality.type = df.snake$mortality.type)

df.disper.snake <- mutate(df.disper.snake, expgroup = revalue(expgroup, c("inoculated" = "Inoculated", 'sham' = 'Sham')))

#

# Model distance to centroid values for different metrics with respect to repeat measures

#

#Jaccard

j1 <- lme(jacc ~ exp.time*expgroup + mortality.type, random = ~ 1 | exp.call , data=df.disper.snake)

j2 <- lme(jacc ~ exp.time*expgroup + mortality.type, random = ~ 1 | exp.call , data=df.disper.snake, correlation = corCAR1(form = ~ exp.time | exp.call))

j3 <- lme(jacc ~ exp.time*expgroup + mortality.type, random = ~ exp.time | exp.call , data=df.disper.snake, correlation = corCAR1(form = ~ exp.time | exp.call))

j4 <- lme(jacc ~ exp.time*expgroup + mortality.type, random = ~ exp.time | exp.call , data=df.disper.snake)

j5 <- lme(jacc ~ exp.time*expgroup + mortality.type, random = ~ 1 | exp.call , data=df.disper.snake, correlation = corCAR1(form = ~ exp.time | exp.call), weights = varFixed(~exp.time))

j6 <- lme(jacc ~ exp.time*expgroup + mortality.type, random = ~ 1 | exp.call , data=df.disper.snake, correlation = corCAR1(form = ~ exp.time | exp.call), weights = varPower(form=~exp.time))

j7 <- lme(jacc ~ exp.time*expgroup + mortality.type, random = ~ 1 | exp.call , data=df.disper.snake, correlation = corCAR1(form = ~ exp.time | exp.call), weights = varExp(form=~exp.time))

j8 <- lme(jacc ~ exp.time*expgroup + mortality.type, random = ~ 1 | exp.call , data=df.disper.snake, correlation = corCAR1(form = ~ exp.time | exp.call), weights = varIdent(form = ~ 1 | expgroup))

AIC(j1,j2,j3,j4,j5, j6, j7, j8) #j2 preferred

summary(j2)

Anova(j2, type=2)

r.squaredLR(j2)[1]*100

#Bray

b1 <- lme(bray ~ exp.time*expgroup + mortality.type, random = ~ 1 | exp.call , data=df.disper.snake)

b2 <- lme(bray ~ exp.time*expgroup + mortality.type, random = ~ 1 | exp.call , data=df.disper.snake, correlation = corCAR1(form = ~ exp.time | exp.call))

b3 <- lme(bray ~ exp.time*expgroup + mortality.type, random = ~ exp.time | exp.call , data=df.disper.snake, correlation = corCAR1(form = ~ exp.time | exp.call), control = lmeControl(maxIter = 1e8, msMaxIter = 1e8, opt = "optim"))

b4 <- lme(bray ~ exp.time*expgroup + mortality.type, random = ~ exp.time | exp.call , data=df.disper.snake)

b5 <- lme(bray ~ exp.time*expgroup + mortality.type, random = ~ 1 | exp.call , data=df.disper.snake, correlation = corCAR1(form = ~ exp.time | exp.call), weights = varFixed(~exp.time))

b6 <- lme(bray ~ exp.time*expgroup + mortality.type, random = ~ 1 | exp.call , data=df.disper.snake, correlation = corCAR1(form = ~ exp.time | exp.call), weights = varPower(form=~exp.time))

b7 <- lme(bray ~ exp.time*expgroup + mortality.type, random = ~ 1 | exp.call , data=df.disper.snake, correlation = corCAR1(form = ~ exp.time | exp.call), weights = varExp(form=~exp.time))

b8 <- lme(bray ~ exp.time*expgroup + mortality.type, random = ~ 1 | exp.call , data=df.disper.snake, correlation = corCAR1(form = ~ exp.time | exp.call), weights = varIdent(form = ~ 1 | expgroup), control = lmeControl(maxIter = 1e8, msMaxIter = 1e8, opt = "optim"))

AIC(b1,b2,b3,b4,b5, b6, b7, b8) #b2 preferred

summary(b2)

Anova(b2, type=2)

r.squaredLR(b2)[1]*100

#raup

r1 <- lme(raup ~ exp.time*expgroup + mortality.type, random = ~ 1 | exp.call , data=df.disper.snake)

r2 <- lme(raup ~ exp.time*expgroup + mortality.type, random = ~ 1 | exp.call , data=df.disper.snake, correlation = corCAR1(form = ~ exp.time | exp.call))

r3 <- lme(raup ~ exp.time*expgroup + mortality.type, random = ~ exp.time | exp.call , data=df.disper.snake, correlation = corCAR1(form = ~ exp.time | exp.call), control = lmeControl(opt = 'optim'))

r4 <- lme(raup ~ exp.time*expgroup + mortality.type, random = ~ exp.time | exp.call , data=df.disper.snake,control = lmeControl(opt = 'optim'))

r5 <- lme(raup ~ exp.time*expgroup + mortality.type, random = ~ 1 | exp.call , data=df.disper.snake, correlation = corCAR1(form = ~ exp.time | exp.call), weights = varFixed(~exp.time))

r6 <- lme(raup ~ exp.time*expgroup + mortality.type, random = ~ 1 | exp.call , data=df.disper.snake, correlation = corCAR1(form = ~ exp.time | exp.call), weights = varPower(form=~exp.time))

r7 <- lme(raup ~ exp.time*expgroup + mortality.type, random = ~ 1 | exp.call , data=df.disper.snake, correlation = corCAR1(form = ~ exp.time | exp.call), weights = varExp(form=~exp.time))

r8 <- lme(raup ~ exp.time*expgroup + mortality.type, random = ~ 1 | exp.call , data=df.disper.snake, correlation = corCAR1(form = ~ exp.time | exp.call), weights = varIdent(form = ~ 1 | expgroup))

AIC(r1,r2,r3,r4,r5, r6, r7, r8) #r2 preferred

summary(r2)

Anova(r2, type=2)

r.squaredLR(r2)[1]*100

# post hoc assessment of interaction using a simple gls

r2.post.control <- gls(raup ~ exp.time, data=filter(df.disper.snake,expgroup == "Sham"), correlation = corCAR1(form = ~ exp.time | exp.call))

summary(r2.post.control)

#

r2.post.inoc <- gls(raup ~ exp.time, data=filter(df.disper.snake,expgroup == "Inoculated"), correlation = corCAR1(form = ~ exp.time | exp.call))

summary(r2.post.inoc)

#

plot(raup ~ exp.time, data=df.disper.snake)

abline(r2.post.control, col = "blue", lty = "dashed")

abline(r2.post.inoc, col = "red")

# Plot multivariate dispersion between groups for different metrics

snake.div.fig1 <- ggplot(df.disper.snake,aes(x=expgroup, y=jacc, shape = expgroup)) + geom_boxplot(outlier.shape=NA, fill = "gray93") + geom_point(position = "jitter", alpha = 0.5, size = 1.5, aes(color = expgroup)) + labs(color = "Treatment", x = "", y = "Distance to centroid", title = "a") + scale_color_brewer(palette = "Set1") + theme(legend.position = "none") + annotate(geom = 'text', label = 'p < 0.001', x = -Inf, y = Inf, hjust = -.1, vjust = 1.2, size = 5)

snake.div.fig2 <- ggplot(df.disper.snake,aes(x=expgroup, y=bray, shape = expgroup)) + geom_boxplot(outlier.shape=NA, fill = "gray93") + geom_point(position = "jitter", alpha = 0.5, size = 1.5, aes(color = expgroup)) + labs(color = "Treatment", x = "", y = "Distance to centroid", title = "b") + scale_color_brewer(palette = "Set1") + theme(legend.position = "none") + annotate(geom = 'text', label = 'p < 0.001', x = -Inf, y = Inf, hjust = -.1, vjust = 1.2, size = 5)

snake.div.fig3 <- ggplot(df.disper.snake,aes(x=exp.time, y=raup, color = expgroup, shape = expgroup)) +

geom_point(alpha = 0.5, size = 1.5) +

geom_smooth(data = filter(df.disper.snake, expgroup == "Inoculated"), method = "lm") +

geom_smooth(data = filter(df.disper.snake, expgroup == "Sham"), method = "lm", se = F, linetype = "dashed") +

labs(color = "Treatment", x = "Time (days)", y = "Distance to centroid", title = "c") +

annotate(geom = 'text', label = 'p = 0.022', x = -Inf, y = Inf, hjust = -1.2, vjust = 10.8, size = 5, color = "#E41A1C") +

annotate(geom = 'text', label = 'p = 0.457', x = -Inf, y = Inf, hjust = -1.3, vjust = 35, size = 5, color = "#377EB8") +

scale_color_brewer(palette = "Set1") +

theme(legend.position = "none")

legend <- get_legend(ggplot(df.disper.snake,aes(x=exp.time, y=raup, color = expgroup, shape = expgroup)) + geom_point(alpha = 0.5, size = 1.5) + geom_smooth(method = "lm") + labs(color = "Treatment", shape = "Treatment", x = "", y = "Distance to Centroid", title = "C") + theme(axis.line = element_line(colour = "black")) + scale_color_brewer(palette = "Set1"))

# Generate multipanel figure that summarizes results of this section

grid.arrange(snake.div.fig1, snake.div.fig2, snake.div.fig3,

legend, ncol=3, nrow = 2,

layout_matrix = rbind(c(1,2,3), c(4,4,4)),

widths = c(2.7, 2.7, 2.7), heights = c(2.5, 0.2))

# 8 - Sham snake mortality effects ####

# richness

df.sham <- df %>%

filter(swab.origin == "snake") %>%

filter(expgroup == "sham") %>%

filter(!is.na(exp.time))

df.sham$week <- factor(df.sham$week, levels = c("w1", "w2", "w3", "w4", "w5", "w6", "w7", "w8", "w9", "w10", "w11", "w12", "w13"))

df.sham$cap.location <- as.factor(df.sham$cap.location)

df.sham$mortality.type <- as.factor(df.sham$mortality.type)

snake.otu <- df.sham[,23:6880]

# GAMM model

# first, evaluate whether to specify random intercepts or random intercepts and slopes

rich.1 <- gam(richness ~ s(exp.time, by = mortality.type, k = 4) + mortality.type + s(week, bs = "re") + s(exp.call, exp.time, bs = 're') + s(mortality.type, bs = "re") + s(cap.location, bs = "re"), family = gaussian(link = "identity"), data = df.sham, select = TRUE, method = "REML")

rich.2 <- gam(richness ~ s(exp.time, by = mortality.type, k = 4) + mortality.type + s(week, bs = "re") + s(exp.call, exp.time, bs = 're'), family = gaussian(link = "identity"), data = df.sham, select = TRUE, method = "REML")

AIC(rich.1,rich.2)

#next assess whether to include an alternative distribution function or transformation link

rich.3 <- gam(richness ~ s(exp.time, by = mortality.type, k = 4) + mortality.type + s(week, bs = "re") + s(exp.call, exp.time, bs = 're'),family = gaussian(link = "log"), data = df.sham, method = "REML")

rich.4 <- gam(richness ~ s(exp.time, by = mortality.type, k = 4) + mortality.type + s(week, bs = "re") + s(exp.call, exp.time, bs = 're'),family = Gamma(link = "identity"), data = df.sham, method = "REML")

rich.5 <- gam(richness ~ s(exp.time, by = mortality.type, k = 4) + mortality.type + s(week, bs = "re") + s(exp.call, exp.time, bs = 're'),family = Gamma(link = "log"), data = df.sham, method = "REML")

rich.6 <- gam(richness ~ s(exp.time, by = mortality.type, k = 4) + mortality.type + s(week, bs = "re") + s(exp.call, exp.time, bs = 're'),family = mgcv::scat(link = "identity"), data = df.sham, method = "REML")

rich.7 <- gam(richness ~ s(exp.time, by = mortality.type, k = 4) + mortality.type + s(week, bs = "re") + s(exp.call, exp.time, bs = 're'),family = mgcv::scat(link = "log"), data = df.sham, method = "REML")

rich.8 <- gam(richness ~ s(exp.time, by = mortality.type, k = 4) + mortality.type + s(week, bs = "re") + s(exp.call, exp.time, bs = 're'),family = poisson(link = "identity"), data = df.sham, method = "REML")

rich.9 <- gam(richness ~ s(exp.time, by = mortality.type, k = 4) + mortality.type + s(week, bs = "re") + s(exp.call, exp.time, bs = 're'),family = poisson(link = "log"), data = df.sham, method = "REML")

AIC(rich.2,rich.3,rich.4,rich.5,rich.6,rich.7,rich.8,rich.9) #rich5 preferred

# next assess whether to include a temporal autocorrelation term

rich.11 <- gam(richness ~ s(exp.time, by = mortality.type, k = 4) + mortality.type + s(week, bs = "re") + s(exp.call, exp.time, bs = 're'),family = Gamma(link = "log"), data = df.sham,correlation = corCAR1(form = ~ exp.time | exp.call), method = "REML")

AIC(rich.5, rich.11)

#Examine most efficient model

summary(rich.5)

k.check(rich.5)

appraise(rich.5, method = "simulate", n_simulate = 1000)

draw(rich.5)

mort.fit <- predict(rich.4, type = "link", newdata = filter(df.sham, mortality.type == "mortality"),

exclude = smooths(rich.4)[4:5])

euth.fit <- predict(rich.4, type = "link", newdata = filter(df.sham, mortality.type == "euthanasia"),

exclude = smooths(rich.4)[4:5])

mort.se <- predict(rich.4, type = "link", newdata = filter(df.sham, mortality.type == "mortality"),

exclude = smooths(rich.4)[4:5], se.fit = T)$se.fit

euth.se <- predict(rich.4, type = "link", newdata = filter(df.sham, mortality.type == "euthanasia"),

exclude = smooths(rich.4)[4:5], se.fit = T)$se.fit

rich.models <- data.frame(

fit = c(mort.fit,euth.fit),

se = c(mort.se, euth.se),

mortality.type = c(rep("Mortality", length(mort.se)), rep("Euthanasia", length(euth.se))),

exp.time = c(filter(df.sham, mortality.type == "mortality")$exp.time, filter(df.sham, mortality.type == "euthanasia")$exp.time),

richness = c(filter(df.sham, mortality.type == "mortality")$richness, filter(df.sham, mortality.type == "euthanasia")$richness)

)

supfig.1 <-ggplot(rich.models, aes(x = exp.time)) +

geom_point(aes(y = richness, color = mortality.type), size = 2, alpha = 0.75) +

geom_smooth(data = . %>% filter(mortality.type == "Euthanasia"), aes(y = fit, color = "Euthanasia"), se = F, linetype = 'longdash') +

geom_smooth(data = . %>% filter(mortality.type == "Mortality"), aes(y = fit, color = "Mortality"), se = F) +

geom_ribbon(data = . %>% filter(mortality.type == "Mortality"), aes(ymax = fit + 1.96*se, ymin = fit - 1.96*se), alpha = 0.2) +

labs(x = "Time (Days)",

y = "OTU Richness",

color = "Mortality Type",

title = "a") +

scale_color_brewer(palette = "Set1") +

annotate(geom = 'text', label = 'p = 0.011', x = -Inf, y = Inf, hjust = -3, vjust = 18, size = 5, color = "#377EB8") +

annotate(geom = 'text', label = 'p = 0.063', x = -Inf, y = Inf, hjust = -1.75, vjust = 40, size = 5, color = "#E41A1C")

# Shannon diversity

# GAMM model

# first, evaluate whether to specify random intercepts or random intercepts and slopes

shan.1 <- gam(shannon ~ s(exp.time, by = mortality.type, k = 4) + mortality.type + s(week, bs = "re") + s(exp.call, exp.time, bs = 're') + s(mortality.type, bs = "re") + s(cap.location, bs = "re"), family = gaussian(link = "identity"), data = df.sham, select = TRUE, method = "REML")

shan.2 <- gam(shannon ~ s(exp.time, by = mortality.type, k = 4) + mortality.type + s(week, bs = "re") + s(exp.call, exp.time, bs = 're'), family = gaussian(link = "identity"), data = df.sham, select = TRUE, method = "REML")

AIC(shan.1,shan.2)

#next assess whether to include an alternative distribution function or transformation link

shan.3 <- gam(shannon ~ s(exp.time, by = mortality.type, k = 4) + mortality.type + s(week, bs = "re")+ s(exp.call, exp.time, bs = 're'),family = gaussian(link = "log"), data = df.sham, method = "REML")

shan.4 <- gam(shannon ~ s(exp.time, by = mortality.type, k = 4) + mortality.type + s(week, bs = "re")+ s(exp.call, exp.time, bs = 're'),family = Gamma(link = "identity"), data = df.sham, method = "REML")

shan.5 <- gam(shannon ~ s(exp.time, by = mortality.type, k = 4) + mortality.type + s(week, bs = "re")+ s(exp.call, exp.time, bs = 're'),family = Gamma(link = "log"), data = df.sham, method = "REML")

shan.6 <- gam(shannon ~ s(exp.time, by = mortality.type, k = 4) + mortality.type + s(week, bs = "re")+ s(exp.call, exp.time, bs = 're'),family = mgcv::scat(link = "identity"), data = df.sham, method = "REML")

shan.7 <- gam(shannon ~ s(exp.time, by = mortality.type, k = 4) + mortality.type + s(week, bs = "re")+ s(exp.call, exp.time, bs = 're'),family = mgcv::scat(link = "log"), data = df.sham, method = "REML")

AIC(shan.2,shan.3,shan.4,shan.5,shan.6,shan.7) #shan6 preferred

# next assess whether to include a temporal autocorrelation term

shan.8 <- gam(shannon ~ s(exp.time, by = mortality.type, k = 4) + mortality.type + s(week, bs = "re")+ s(exp.call, exp.time, bs = 're'),family = mgcv::scat(link = "identity"), data = df.sham,correlation = corCAR1(form = ~ exp.time | exp.call), method = "REML")

AIC(shan.6, shan.8)

summary(shan.6)

k.check(shan.6)

appraise(shan.6, method = "simulate", n_simulate = 1000)

draw(shan.6)

mort.fit <- predict(shan.6, type = "link", newdata = filter(df.sham, mortality.type == "mortality"),

exclude = smooths(shan.6)[4:5])

euth.fit <- predict(shan.6, type = "link", newdata = filter(df.sham, mortality.type == "euthanasia"),

exclude = smooths(shan.6)[4:5])

mort.se <- predict(shan.6, type = "link", newdata = filter(df.sham, mortality.type == "mortality"),

exclude = smooths(shan.6)[4:5], se.fit = T)$se.fit

euth.se <- predict(shan.6, type = "link", newdata = filter(df.sham, mortality.type == "euthanasia"),

exclude = smooths(shan.6)[4:5], se.fit = T)$se.fit

shan.models <- data.frame(

fit = c(mort.fit,euth.fit),

se = c(mort.se, euth.se),

mortality.type = c(rep("Mortality", length(mort.se)), rep("Euthanasia", length(euth.se))),

exp.time = c(filter(df.sham, mortality.type == "mortality")$exp.time, filter(df.sham, mortality.type == "euthanasia")$exp.time),

shannon = c(filter(df.sham, mortality.type == "mortality")$shannon, filter(df.sham, mortality.type == "euthanasia")$shannon)

)

supfig.2 <- ggplot(shan.models, aes(x = exp.time)) +

geom_point(aes(y = shannon, color = mortality.type), size = 2, alpha = 0.75) +

geom_smooth(data = . %>% filter(mortality.type == "Euthanasia"), aes(y = fit, color = "Euthanasia"), se = F, linetype = 'longdash') +

geom_smooth(data = . %>% filter(mortality.type == "Mortality"), aes(y = fit, color = "Mortality"), se = F) +

geom_ribbon(data = . %>% filter(mortality.type == "Mortality"), aes(ymax = fit + 1.96*se, ymin = fit - 1.96*se), alpha = 0.2) +

labs(x = "Time (Days)",

y = "Shannon Diversity",

color = "Mortality Type",

title = "b") +

scale_color_brewer(palette = "Set1") +

annotate(geom = 'text', label = 'p = 0.001', x = -Inf, y = Inf, hjust = -3, vjust = 4, size = 5, color = "#377EB8") +

annotate(geom = 'text', label = 'p = 0.070', x = -Inf, y = Inf, hjust = -1.6, vjust = 25, size = 5, color = "#E41A1C")

# Shannon evenness

# calculate shannon evenness

df.sham$evenness <- exp(df.sham$shannon)/(df.sham$richness)

# GAMM model

# first, evaluate whether to specify random intercepts or random intercepts and slopes

even.1 <- gam(evenness ~ s(exp.time, by = mortality.type, k = 4) + mortality.type + s(week, bs = "re") + s(exp.call, exp.time, bs = 're') + s(mortality.type, bs = "re") + s(cap.location, bs = "re"), family = gaussian(link = "identity"), data = df.sham, select = TRUE, method = "REML")

even.2 <- gam(evenness ~ s(exp.time, by = mortality.type, k = 4) + mortality.type + s(week, bs = "re") + s(exp.call, exp.time, bs = 're'), family = gaussian(link = "identity"), data = df.sham, select = TRUE, method = "REML")

AIC(even.1,even.2)

#next assess whether to include an alternative distribution function or transformation link

even.3 <- gam(evenness ~ s(exp.time, by = mortality.type, k = 4) + mortality.type + s(week, bs = "re")+ s(exp.call, exp.time, bs = 're'),family = gaussian(link = "log"), data = df.sham, method = "REML")

even.4 <- gam(evenness ~ s(exp.time, by = mortality.type, k = 4) + mortality.type + s(week, bs = "re")+ s(exp.call, exp.time, bs = 're'),family = Gamma(link = "identity"), data = df.sham, method = "REML")

even.5 <- gam(evenness ~ s(exp.time, by = mortality.type, k = 4) + mortality.type + s(week, bs = "re")+ s(exp.call, exp.time, bs = 're'),family = Gamma(link = "log"), data = df.sham, method = "REML")

even.6 <- gam(evenness ~ s(exp.time, by = mortality.type, k = 4) + mortality.type + s(week, bs = "re")+ s(exp.call, exp.time, bs = 're'),family = mgcv::scat(link = "identity"), data = df.sham, method = "REML")

even.7 <- gam(evenness ~ s(exp.time, by = mortality.type, k = 4) + mortality.type + s(week, bs = "re")+ s(exp.call, exp.time, bs = 're'),family = mgcv::scat(link = "log"), data = df.sham, method = "REML")

AIC(even.2,even.3,even.4,even.5,even.6,even.7)

#even2 preferred

# next assess whether to include a temporal autocorrelation term

even.8 <- gam(evenness ~ s(exp.time, by = mortality.type, k = 4) + mortality.type + s(week, bs = "re")+ s(exp.call, exp.time, bs = 're'),family = gaussian(link = "identity"), data = df.sham,correlation = corCAR1(form = ~ exp.time | exp.call), method = "REML")

AIC(even.2, even.8)

summary(even.2)

appraise(even.2, method = "simulate", n_simulate = 1000)

draw(even.2)

mort.fit <- predict(even.2, type = "link", newdata = filter(df.sham, mortality.type == "mortality"),

exclude = smooths(even.2)[4:5])

euth.fit <- predict(even.2, type = "link", newdata = filter(df.sham, mortality.type == "euthanasia"),

exclude = smooths(even.2)[4:5])

mort.se <- predict(even.2, type = "link", newdata = filter(df.sham, mortality.type == "mortality"),

exclude = smooths(even.2)[4:5], se.fit = T)$se.fit

euth.se <- predict(even.2, type = "link", newdata = filter(df.sham, mortality.type == "euthanasia"),

exclude = smooths(even.2)[4:5], se.fit = T)$se.fit

even.models <- data.frame(

fit = c(mort.fit,euth.fit),

se = c(mort.se, euth.se),

mortality.type = c(rep("Mortality", length(mort.se)), rep("Euthanasia", length(euth.se))),

exp.time = c(filter(df.sham, mortality.type == "mortality")$exp.time, filter(df.sham, mortality.type == "euthanasia")$exp.time),

evenness = c(filter(df.sham, mortality.type == "mortality")$evenness, filter(df.sham, mortality.type == "euthanasia")$evenness)

)

supfig.3 <- ggplot(even.models, aes(x = exp.time)) +

geom_point(aes(y = evenness, color = mortality.type), size = 2, alpha = 0.75) +

geom_smooth(data = . %>% filter(mortality.type == "Euthanasia"), aes(y = fit, color = "Euthanasia"), se = F, linetype = 'longdash') +

geom_smooth(data = . %>% filter(mortality.type == "Mortality"), aes(y = fit, color = "Mortality"), se = F, linetype = 'longdash') +

labs(x = "Time (Days)",

y = "Shannon Evenness",

color = "Mortality Type",

title = "c") +

scale_color_brewer(palette = "Set1") +

annotate(geom = 'text', label = 'p = 0.157', x = -Inf, y = Inf, hjust = -3, vjust = 14, size = 5, color = "#377EB8") +

annotate(geom = 'text', label = 'p = 0.797', x = -Inf, y = Inf, hjust = -3, vjust = 37, size = 5, color = "#E41A1C")

supfig.3

# Generate summary figure

supfig <- ggarrange(supfig.1, supfig.2, supfig.3, common.legend = T, legend = "bottom", ncol = 3)

supfig
