# Supplementary figures and images for: Host microbiome responses to the Snake Fungal Disease pathogen (Ophidiomyces ophidiicola) are driven by changes in microbial richness
Source: Sci Rep. 2022 Feb 23;12:3078. doi: 10.1038/s41598-022-07042-5 (PMC8866498; doi:10.1038/s41598-022-07042-5)

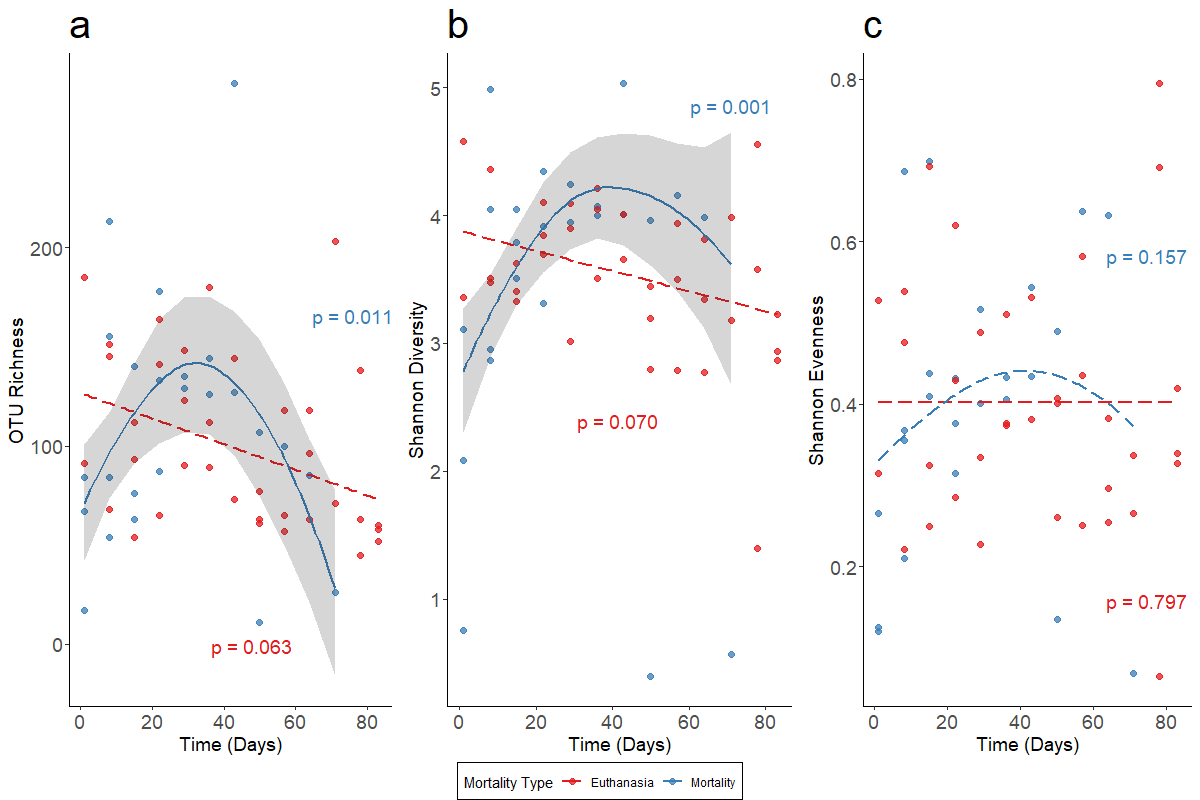

Supplement: Supplementary file 4 — Supplementary Information 4. [file 41598_2022_7042_MOESM4_ESM.tiff]
